# Proteomic analysis of the marine diatom *Thalassiosira pseudonana *upon exposure to benzo(a)pyrene

**DOI:** 10.1186/1471-2164-12-159

**Published:** 2011-03-24

**Authors:** Raquel N Carvalho, Teresa Lettieri

**Affiliations:** 1 European Commission - Joint Research Centre, Institute for Environment and Sustainability, Rural, Water, and Ecosystem Resources Unit, T.P. 270, Via E. Fermi 2749, 21027 Ispra (VA), Italy

## Abstract

**Background:**

Polycyclic aromatic hydrocarbons (PAHs) are environmental pollutants ubiquitously distributed. They are generated by incomplete combustion of organic materials such as wood or fossil fuels. Due to their carcinogenic, mutagenic effects and to their wide distribution in the environment, these pollutants pose many concerns to researchers and regulators. In our laboratories we investigated the effect of benzo(a)pyrene (BaP) exposure in the marine diatom *Thalassiosira pseudonana*, which has become an important model organism in aquatic toxicology studies.

**Results:**

In order to investigate the mechanism of action of PAHs, we exposed the diatoms for 24 h to 36.45 μg/L of BaP which inhibits the growth by about 30%, and analysed the relative protein expression profile by a quantitative proteomics approach based on iTRAQ labels. The proteomics profile analysis showed that around 10% of the identified proteins were regulated and one fourth of them confirmed the gene expression changes seen by DNA microarray. Particularly interesting was the down regulation of the Silicon transporter 1 (SIT1), an enzyme that is responsible for the uptake of silicon from the media into the diatom cells. Regulation of SIT1 upon BaP treatment was also confirmed at the gene expression level.

**Conclusions:**

The potential use of the regulated proteins found in this study as early indicators of environmental exposure to PAHs is discussed. In particular, SIT1 is considered a promising biomarker and SIT1 expression changes were confirmed also when the diatoms were exposed to field samples, e.g. marine surface sediments contaminated by PAHs.

## Background

Diatoms are eukaryotic, unicellular, photosynthetic organisms that inhabit marine and fresh waters worldwide and are responsible for about 40% of the total carbon fixation in oceans [1]. Their high productivity compared to other primary producers is likely the main base of the world's marine food webs, allowing a reduced trophic fractionation, and sustaining even top-level marine predators like cetaceans [2]. Diatoms are surrounded by a peculiar cell wall with intricate micro- and nano-structured biosilica patterns. The assembly of specific silica shell patterns for the different diatom species is under genetic control, being reliably reproduced on each cell division [3]. *Thalassiosira pseudonana *is a centric diatom inhabiting marine ecosystems which has been a target organism for studies focusing on diatom biosilica formation [4,5], the molecular effects of copper toxicity [6] and nutrient limitation, temperature or pH effects [7]. In 2004, the whole genome of *T. pseudonana *was published [8] and soon after, the genome sequencing of two other diatom organisms has been described [1,9]

The available genomic information for these diatom species, associated with the important role they play in the carbon fixation in oceans, paved the way for their use as model organisms in molecular ecotoxicology studies. Thus, our laboratory has designed a DNA microarray chip containing all the available genes in *T. pseudonana *to identify gene biomarkers of exposure to stress conditions, including environmental pollutants. Some pollutants which have been initially selected are the polycyclic aromatic hydrocarbons (PAHs). PAHs are persistent organic pollutants with a ubiquitous distribution in aquatic environments and are considered a major threat to marine and freshwater ecosystems [10]. Several PAHs were included in the priority list of the European Union's Water Framework Directive (2000/60/EC) because of their potential carcinogenic and mutagenic properties. Previously, an axenic culture of *T. pseudonana *has been used to investigate the molecular effects of exposure to three polycyclic aromatic hydrocarbons (PAHs), both as single compounds and as a mixture [11]. By studying the expression of selected genes by quantitative real-time PCR (qRT-PCR), a few genetic biomarkers of exposure to PAHs have been described [11]. Recently, we have extended the gene expression analysis to the entire *T. pseudonana *transcriptome by using our in-house microarray for exposure experiments to benzo(a)pyrene (BaP), a common PAH compound. The regulated genes identified by DNA microarray increase our understanding of the pathways involved in the cellular response to PAH exposure conditions and allow the selection of some interesting genes to be used as molecular biomarkers of exposure to PAHs in the environment. For this purpose, we have recently tested diatoms exposed to contaminated marine water sediments and confirmed the suitability of some genes as biomarkers in environmental monitoring studies [12].

Recent advances in shotgun proteomics and the use of isobaric labelling of peptides and proteins for the relative quantification of an entire organism or cell proteome under different experimental conditions offer promising tools for the discovery of biomarkers also at the protein level. We describe here a procedure for proteomic analysis of the diatom *T. pseudonana *using iTRAQ labelling to identify proteins differentially expressed upon exposure to BaP. The data obtained will be discussed in the context of the gene expression analysis performed in our laboratory under the same experimental conditions, as well as to the known effects of PAHs in other organisms. Ultimately, our goal is to use the molecular information gathered in this model organism, as indicators for the early assessment of the ecosystem health and predict the effects in higher eukaryotes of the same habitats. These molecular indicators alone or in combination with the common chemical analysis of pollutants in the environment should provide a better understanding of the endangerment level of an ecosystem to specific stress conditions.

## Results

### Effect of BaP on diatom growth

**Figure 1:**
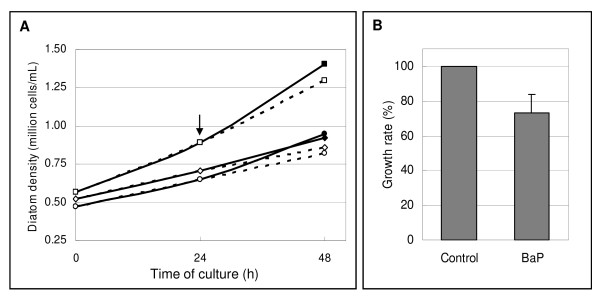
**Growth curve of diatom cultures**. (A) Line and scatter graph shows the diatom culture density along culture time in BaP-exposed (open symbols, dashed line) and control (closed symbols, continuous line) conditions, in three biological replicates. The black arrow indicates the time when exposure started. (B) Vertical graph shows the diatom average decrease in the growth rate caused by BaP treatment after 24 h exposure, when compared to control conditions; error bars represent the standard deviation among the three biological replicates.

To study the effects of BaP exposure in diatoms at the molecular level, *T. pseudonana *cultures have been exposed for 24 h to 36.45 μg/L BaP or just to the methanol solvent (here referred to as control conditions). Methanol at a final concentration of 0.05% (v/v) was shown to induce no effect on the diatom growth. In control conditions, diatom cell density showed an increase by 1.5 fold after 24 h (Figure 1). Exposure of diatom cultures to BaP for 24 h resulted in 30% growth inhibition relative to the control (Figure 1), which is consistent with what had been previously described [11].

### Protein identification

Protein extraction from three biological replicates, each consisting of both control and BaP-exposed conditions, was carried out on consecutive days. Additional file 1 shows the workflow followed in the present study, starting from the cell culture and exposure to BaP up to MS/MS data analysis. The total amount of protein obtained was around 2 μg/million cells harvested from the control conditions, as compared with 1.5 μg/million cells extracted from the BaP-exposed conditions. Simultaneous protein measurements in all the six samples (See Methods section) were performed to assure that the same amount of protein from each sample was taken for iTRAQ labeling. In addition, both undigested and tryptic-digested samples were loaded on a SDS-PAGE to confirm both the protein amount and the complete digestion by trypsin (Additional file 2). Following SCX separation of the pooled iTRAQ-labeled samples and LC-MS/MS analysis, we were able to identify a total of 308 different proteins in the three biological replicates, based on one or more peptide hits per protein at >95% confidence. ProteinPilot was used to search the complete *T. pseudonana *proteome database downloaded from UniProtKB/Swiss-Prot. Following exclusion of proteins identified by a single peptide, the number of proteins identified decreased to 200 (Additional file 3: Table S1). The false discovery rate of peptide matches above identity threshold was 0.58%. Based on gene ontology classification, the major group of proteins identified is involved in translation, metabolic processes and photosynthesis (Additional file 3: Table S1). In addition, about 20% of the proteins identified in this study had no annotated function assigned to them.

### Protein quantification

iTRAQ-mediated shotgun proteomics allows the relative quantification of peptides from up to eight samples simultaneously, and has proven to be a successful tool in protein biomarker discovery, both in the biomedical [13,14] and environmental fields [15,16].

**Figure 2:**
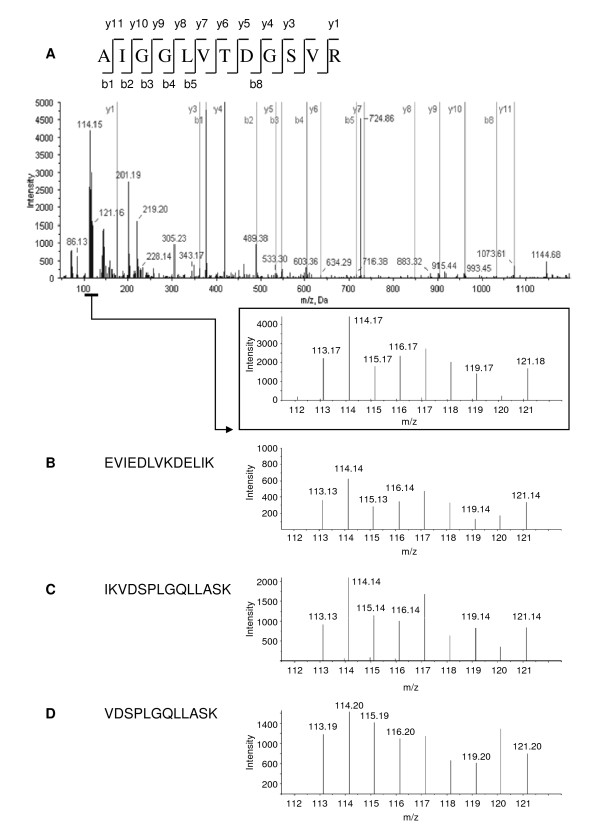
**An example of tandem mass spectrometry spectra that exhibited significant iTRAQ ratios between BaP-exposed and control conditions**. (A) The fragmentation spectrum of AIGGVTDGSVR, a peptide fragment from predicted C-5 cytosine-specific DNA methylase (B8BVP1) is shown. The reporter ion signals appear in the low-mass region of the spectrum and are used to determine the relative amount of the protein in *T. pseudonana *exposed to BaP (iTRAQ 114, 116 and 121) when compared to control conditions (iTRAQ 113, 115 and 119) (inset). (B-D) Reporter ion signals for other peptide fragments of B8BVP1.

Different iTRAQ reagents were used to distinguish peptides obtained from diatom cells cultured in control and BaP-exposed conditions (Additional file 1). Protein quantification based on the relative amounts of the different iTRAQ labels was obtained for all identified proteins based on two or more unique peptides, using both ProteinPilot and Mascot algorithms. The use of both algorithms allowed the identification of a higher number of regulated proteins, because of slight differences in peptide confidence levels and iTRAQ-label quantification values acquired with the two programs. We then analyzed the data sets for changes in protein levels upon BaP-exposure. The expression of a protein was considered as being regulated by BaP treatment when it showed changes in expression beyond 20% (i.e. >1.2- or <0.8-fold change from unity; calculated from the median ratio of all reported proteins), and with a *P *< 0.05 calculated after a two-tailed unpaired statistical approach. Following these criteria, 13 different proteins with two or more quantified peptides, showed regulation upon BaP exposure. As listed in Table 1, 6 proteins showed an up-regulation and 7 proteins a down-regulation. In addition, for those proteins with a significant regulation of at least 20% (*P *< 0.05), but identified by a single peptide at >95% confidence, and for which other peptides had been identified at lower confidence, all individual spectra were analyzed manually, both for the fragmentation pattern and the relative intensity of the different iTRAQ reporter ions. Six proteins which showed a significant regulation considering all the peptides (*P *< 0.05) were selected for inclusion in the potentially BaP-regulated proteins (Table 1; Additional file 4: Supplemental Figures S1-S6). Our decision to include these proteins in Table 1 as "potentially regulated" is sustained by the risk of loosing otherwise important protein targets for follow-up studies, masked by the high variability existing in unsynchronized diatom cultures. It is even more relevant since for two of them, predicted NonF-related protein; DJ-1/PfpI family (Swiss-Prot:B5YN08) and silicon transporter 1 (Swiss-Prot:Q0QVM8), a similar regulation has been confirmed at the gene expression level, by DNA microarray (unpublished data). The regulated proteins were mainly involved in metabolism, photosynthesis and transport activities (Table 1). Figure 2 depicts an example of protein identification and quantification for a predicted C-5 cytosine-specific DNA methylase (Swiss-Prot:B8BVP1). The b- and y- ion series in the tandem mass spectrometry spectra were used to identify the peptides. The iTRAQ reporter ion peak areas of the four peptide fragments (AIGGVTDGSVR, EVIEDLVKDELIK, IKVDSPLGQLLASK and VDSPLGQLLASK, Figure 2A inset, B-D) were used to measure the relative amount of C-5 cytosine-specific DNA methylase in BaP-exposed (at m/z 114, 116 and 121) and control diatom cultures (at m/z 113, 115 and 119).

**Table 1:** Changes in the relative abundance of proteins in *T. pseudonana *exposed to benzo(a)pyrene

Regulated proteins based on two or more peptides at >95% confidence						
**Name**	**Accession^a^**	**Gene name**	**GO**	**Peptides** **(95%)^b^**	**Regulation** **Factor^c^**	**t-test**

C-5 cytosine-specific DNA methylase, P	B8BVP1	THAPSDRAFT_21517	Metabolic process	4	2.30	0.045
Mitochondrial chaperonin	B5YLQ5^d^	HSP60	Chaperone	7	1.48	0.018
Cysteine synthase	B8BUL0	THAPSDRAFT_270338	Biosynthetic process	2	1.30	0.003
N-acetylornithine aminotransferase	B8CGH5^d^	THAPSDRAFT_270136	Metabolic process	3	1.28	0.004
3-isopropylmalate dehydrogenase	B8C2I2	THAPSDRAFT_268875	Oxidation reduction	3	1.21	0.001
Proteasome subunit alpha type	B8C0E7^d^	PSA4	Protein catabolism	2	1.21	0.032
Fucoxanthin chl a/c light-harvesting protein	B8C2Y4	Lhcr10	Photosynthesis	2	0.76	0.021
Eukaryotic translation initiation factor 4A	B8CC33	THAPSDRAFT_9716	Translation	2	0.68	0.041
CPSase	B8BZG0	THAPSDRAFT_40323	Metabolic process	5	0.63	0.007
Predicted protein	B8C0M3	THAPSDRAFT_22483	Unassigned	2	0.55	0.041
Predicted long chain acyl CoA synthetase	B8CDL6	THAPSDRAFT_25042	Metabolic process	2	0.53	0.050
Fucoxanthin chl a/c light-harvesting protein	B8C0K3	Lhcr4	Photosynthesis	2	0.50	0.003
Adenine nucleotide translocator	B8BR30	ANT1	Membrane transport	3	0.45	0.049

**Potentially regulated proteins based on a single peptide at >95% confidence and additional peptides with lower confidence**						

**Name**	**Accession**	**Gene name**	**GO**	**Peptides** **(95%)**	**Regulation** **factor**	**t-test**

Dihydrodipicolinate reductase	B8C5E0	THAPSDRAFT_28544	Oxidation reduction	1	1.67	0.030
Predicted NonF-related protein; DJ-1/PfpI family	B5YN08^d^	THAPS_6979	Unassigned	1	1.21	0.016
Inorganic pyrophosphatase	B8C6T9	THAPSDRAFT_269348	Metabolic process	1	1.21	0.030
Porin/voltage-dependent anion-selective channel	B8BQH4	THAPSDRAFT_25918	Membrane transport	1	0.80	0.043
Silicon transporter	Q0QVM8^d^	SIT1	Membrane transport	1	0.78	0.013
Rubisco expression protein	B8LEU2	cfxX	Photosynthesis	1	0.57	0.041

^a ^Accession numbers, gene name and gene ontology information of proteins taken from the UniProtKB/Swiss-Prot database

^b ^Values indicate the number of peptides used for identification and quantification of the proteins.

^c ^Protein ratios in three biological replicates with 0.8 > Regulation factor < 1.2 and P-values <0.05

^d ^Proteins showing the same regulation trend in the DNA microarray experiments

As expected, the quantification of iTRAQ labels 113, 114, 115, 116, 119 and 121 cluster on a principle component analysis (PCA) according to the conditions of exposure (Additional file 5), i.e. control (113, 115 and 119) vs BaP-exposed (114, 116 and 121). In addition, the quantification of iTRAQ labels 117 and 118 cluster together on the PCA and away from all other labels (Additional file 5).

iTRAQ labels 117 and 118 have been used to label untreated and BaP-exposed conditions, respectively on an old diatom culture which was in resting phase. Thus, when we subjected this culture to the same experimental procedure, no significant growth could be observed in the control and BaP-exposed cultures after 24 h. This prevented the measurement of any BaP-induced effect either as growth inhibition or as a difference in expression of the proteome, which underlines the importance of using cell cultures in exponential growth for the purpose of molecular biomarker discovery.

### Gene expression analysis of SIT1

**Figure 3:**
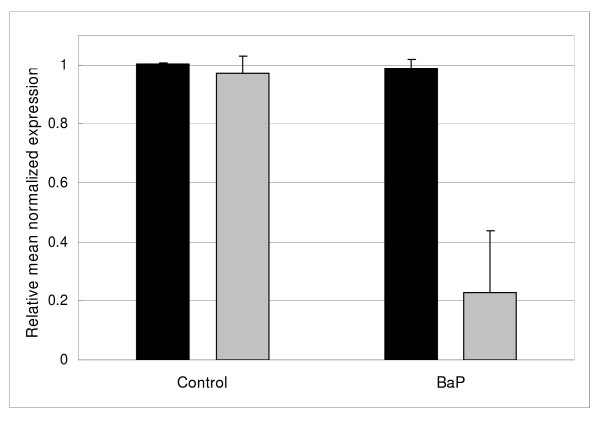
**Silicon transporter 1 gene regulation**. Bar graph shows the normalized expression mean of the *sit1 *gene (gray) relative to the expression of a housekeeping gene *gapdh *(black) by qRT-PCR. Total RNA extracted from benzo(a)pyrene-exposed (BaP) and control diatom cultures has been tested for a total of 6 different biological replicates. The bars represent the standard deviation among the different replicates.

The identification of the silicon transporter (SIT1) in our study as a potentially regulated protein after BaP-treatment (Table 1) was a very interesting finding, since BaP and other polycyclic aromatic hydrocarbons have been previously shown to regulate the gene expression of several silaffin proteins[11]. Silaffins are involved in the silica biomineralization of diatoms [17] and rely on the availability of the silicon pool inside the cells, gathered through uptake from the environment by silicon transporters located at the membrane. In order to confirm the effect of BaP on the silicon uptake by SIT1 in *T. pseudonana*, suggested from the present proteomics study, we have designed specific primers to study the gene expression of this transporter by qRT-PCR. Consistent with the proteomics results, also the expression of the *sit1 *gene showed a down-regulation in all the six biological replicates for BaP exposure tested (Figure 3). In addition, when diatom cultures were exposed to a mixture of PAHs extracted from an environmental contaminated site (port of Genoa, Italy), with a BaP concentration 20-fold lower as the one used in the present study, the expression of the *sit1 *gene still showed a decrease by qRT-PCR [12].

## Discussion

Since the publication of the entire genome sequence of *T. pseudonana *in 2004 [8], this eukaryotic algae has become a promising model organism to study stress responses in marine environments at the molecular level. Diatom species are particularly relevant as model organisms because i) they are unicellular organisms easy to keep in culture, ii) they have a predominant role as primary producers, accounting for about 40% of the total carbon fixation in oceans and iii) any fluctuation at the population level of these organisms is expected to have major effects on the entire ecosystem [1]. In this study, diatom cultures have been exposed to BaP, a common PAH compound with carcinogenic and mutagenic properties, and found in several fresh water and salt water habitats, arising from various natural and anthropogenic sources. The major areas of concern for these pollutants are the estuarine, coastal zones, port areas and also enormous marine environmental areas due to navigation activities as well as to oil spill accidents. A previous report from our group had already unveiled some biological pathways involved in the diatom response to BaP exposure, from the expression analysis of selected genes by qRT-PCR, including lipid metabolism and the biosilicification process [11]. The current report is part of a broader effort to identify biomarkers of exposure at both gene and protein expression level. Currently, even with the available genome sequence of *T. pseudonana*, a deep understanding of the pathways involved in any response to stress conditions is hindered by the limited amount of information on the protein function. In fact, the available annotation on the function or metabolic pathways of most of the *T. pseudonana *proteins has been mainly deduced from sequence homology with other organisms and many are only predicted or putative proteins with no function assigned. So far, only few studies describing the function of selected proteins have been published e.g. the ones involved in the silicon uptake and biomineralization [17] and specific cell-surface proteins potentially involved in the copper-induced stress response [6,18]. The need for comprehensive proteomics studies is therefore relevant for the purpose of compiling the entire translated pool of genes in this organism and to allow the build-up of metabolic or signaling pathways that could be involved in stress and adaptation-related responses. Mass spectrometry analyses of *T. pseudonana *for the purpose of protein identification have previously been performed on a cell wall fraction, identifying a total of 31 proteins [19] and recently on the whole-cell proteome, with the identification of 1928 different proteins [20]. To our knowledge, the present data describe the first use of a quantitative proteomics approach in *T. pseudonana*, to identify potential protein biomarkers of exposure to stress conditions in this organism. However, only about 2% of the entries on the predicted diatom proteome database have been identified in this study, suggesting that further development of extraction and fractionation techniques are required to improve the identification of low abundant or membrane proteins. Interestingly, around 14% of the identified proteins and 10% of the regulated proteins found in this study are annotated as predicted or putative proteins, which limits the assignment of the pathways involved in the response to BaP exposure.

### Photosynthesis

Three different proteins involved in photosynthetic processes show a down-regulation in BaP-exposed samples, compared to control conditions. This finding is in agreement with previous reports on a harmful effect of BaP and other PAHs on the photosynthetic activity in plants [21]. One can speculate that a reduction in the diatoms photosynthesis caused by a PAH contamination in oceans could result in a lower consumption of atmospheric carbon dioxide by diatoms and decrease the generated organic carbon. Because diatoms are the contemporaneous dominant primary producers in marine environments, a reduction in their activity is likely to affect the marine food webs they sustain [1,2].

### DNA methylation

Gene methylation is an ancient property of eukaryotic genomes [22] and is a source of epigenetic information, by providing instructions on how, where, and when the genetic information should be transcribed [23]. In mammalian cells, cytosine-specific methyltransferases methylate CpG sequences, which are believed to modulate gene expression and cell differentiation [24]. In the present study, a predicted C-5 cytosine-specific DNA methylase, an enzyme which specifically methylates the C-5 carbon of cytosines in DNA to produce C5-methylcytosine, revealed an up-regulation in the BaP-exposed diatom cultures compared to control conditions. This finding is consistent with the recent DNA microarray studies performed in our laboratory, where another putative DNA methyltransferase also showed an up-regulation of gene expression (data not shown). Interestingly, variable degrees of overexpression of DNA methyltransferases have been reported in tumor tissue [25]. Changes in the DNA methylation patterns in tumor cells often involve global hypomethylation of the genome accompanied by region-specific hyper-methylation events [23]. In addition, exposure to BaP has been connected with a disruption in DNA methylation in cancerous cells [26,27]. Therefore, alterations at the level of DNA methylation caused by BaP exposure could be linked with the carcinogenic properties of this compound. In addition, this effect seems to be conserved in eukaryotes, strengthening further the use of *T. pseudonana *as a model organism to study general toxicity events.

### Lipid metabolism

Due to its hydrophobic nature, PAHs accumulate in lipidic environments such as membranes and are known to induce oxidative stress and lipid peroxidation [28]. From the candidate biomarkers found in the present study, the regulated long chain acyl CoA synthetase protein (Swiss-Prot:B8CDL6) is involved in lipid metabolism, most likely belonging to the long-chain acyl CoA synthetase family of enzymes, as deduced from sequence homology to other organisms. Although there are no available studies on the catalytic activity of long-chain acyl-CoA synthetase in diatoms; in mammals, they catalyze the formation of long-chain CoA and AMP from fatty acid, CoA and ATP, in a two step reaction that initiates cellular long-chain fatty acid metabolism [29]. In this study, the long chain acyl CoA synthetase protein shows a 2-fold down-regulation upon exposure to BaP. A similar down-regulation has been observed for an enzyme belonging to this family by DNA microarray studies in adult zebrafish exposed to produced water components, which are rich in PAHs [30]. In *T. pseudonana*, there are six predicted proteins belonging to the long-chain acyl-CoA synthetase family [8]. The regulation of this pathway is further evidenced in a previous report that showed a regulation of another protein belonging to this family, the long-chain-fatty-acid--CoA ligase (Swiss-Prot:B8CBL9) in the opposite direction. Thus, an increased expression upon exposure to BaP has been observed at the gene level, both by qRT-PCR [11] and by DNA microarray, as well as at the protein level, as seen by immunoassays[12].

The involvement of the lipid metabolism on the cellular response to BaP exposure is further evidenced on a recent report in human macrophages where it was demonstrated that BaP is able to trigger lipid accumulation in cellular compartments, including endosomes and lysosomes [31]. We can speculate that such increase in lipids could be involved in the more efficient sequestering of the hydrophobic BAP, thus preventing the formation of DNA adducts generated by BaP metabolites. In mammalian cells, five different long-chain acyl-CoA synthetase proteins have been described, with specific and sometimes overlapping substrates and subcellular localizations [32]. Further studies would be necessary to uncover the potential functional differences, including substrate specificities, of this family of enzymes in the diatom species, which are likely to be regulated in different ways by BaP exposure. Despite the mechanism of action involved, it is clear that the cellular response to PAHs in *T. pseudonana *include the regulation of enzymes involved in lipid metabolism, in particular long-chain fatty acids. In addition, since this family of enzymes seems to be conserved across all phylogenetic branches, it is possible that a similar regulation may take place in other organisms.

### Silicon uptake and metabolism

One characteristic feature of diatoms is the silicified cell-wall or frustule that surrounds the plasma membrane [33] which is assembled by the combined action of silaffin proteins and long-chain polyamines (LCPA). Silaffin 1-2L and Silaffin 3 in *T. pseudonana *have been shown to directly catalyze silica polymerization and the activity of the different proteins determines the size and shapes of produced silica particles, as demonstrated from scanning electron microscopy analysis of distinct silaffin-LCPA mixtures [17]. Maintenance of the diatom intracellular silicon pools requires the uptake of silicon from the aquatic environment through membrane silicon transporters. Silicon transporter 1 (SIT1) and silicon transporter 2 (SIT2) have been previously shown to be associated with cell cycle events, with a gene expression peak in the S phase, concomitant with DNA replication and cytokinesis [34]. The increase in SIT expression likely raises the uptake of silicon from the environment, preceding the synthesis of new valves in the silica deposition vesicle (SDV) and exocytosis in the G2+M phase [35].

In *T. pseudonana *cultures exposed to BaP, we observe a down-regulation of SIT1 (Swiss-Prot:Q0QVM8), both at the protein level and gene expression level. Interestingly, BaP treatment also cause a reduction in the net silicon uptake per diatom cell, as determined by the measurement of silicon disappearance from the medium [12]. This finding suggests a direct link between BaP treatment and a decrease in the available intracellular pools of silicon, presumably through a decrease in expression of SIT1. Despite the strong amino acid identity between SIT1 and SIT2 (88%) [34], the peptides identified in this study for SIT1 correspond to regions not identical to both enzymes. Therefore, no conclusion can be drawn on whether SIT2 expression is also affected by BaP treatment. Previously, silicon limitation was shown to specifically down-regulate silaffin 3 expression [7], as we also observe upon BaP treatment [11,12]. It would be interesting to test whether the combined down-regulation of SIT1, the intracellular silicon limitation conditions, and the down regulation of Sil3 caused by BaP could affect the silicification process and the produced silica shell nanopatterns in *T. pseudonana*. For this purpose, it is likely that higher times of exposure than the 24 h used in the present study, and the establishment of cell synchronization prior to exposure would be required for the visualization of changes at the level of cell wall morphology by e.g. scanning electron microscopy (SEM).

## Conclusions

Using a quantitative proteomics approach based on iTRAQ labeling of peptides, we could study protein expression changes in diatoms upon exposure to BaP. Despite the low protein identification rate achieved in our studies, we were able to select a set of 19 proteins with changes in expression. Our data shows that the lipid metabolism and biosilicification processes are involved, as observed previously, but also unveils other potential pathways involved in the diatom exposure or in toxicity responses to BaP, such as DNA methylation and photosynthesis. These data, combined with gene expression analysis can help to elucidate pathway/metabolic processes involved in the mode of action of pollutants.

## Methods

### Diatom culture and detection of growth inhibition upon exposure to benzo(a)pyrene

*T. pseudonana *(strain CCMP 1335) was obtained as axenic culture from the Provasoli-Guillard National Center for Culture of Marine Phytoplankton (CCMP, West Boothbay Harbour, Maine, USA). Diatoms were maintained at 6-8°C under a diurnal light cycle of 13 h light and 11 h darkness. The culture medium was f/2-medium based on 3.2% artificial sea water (ASW, Sigma-Aldrich, Steinheim, Germany). *T. pseudonana *was cultured at densities between 0.5 × 10^6 ^and 2 × 10^6 ^cells/mL. Fresh cultures for maintenance were inoculated every 7 days. Cell densities were determined by measuring the absorption at 450 nm using a microplate spectrophotometer (Biorad, Hercules, CA, USA) and used to calculate growth rates, as previously described [11].

Fresh diatom cultures were inoculated at a start cell density of 0.5 × 10^6 ^cells/mL, incubated for 24 h and divided into different flasks for exposure experiments. Diatom cultures were exposed to benzo(a)pyrene (36.45 μg/L) dissolved in methanol while the solvent was added to control cultures at a final concentration of 0.05% (v/v) [11], half-hour before the beginning of the light cycle. After 24 h of exposure, and before the beginning of the light cycle, cell densities were measured and growth rates were used to determine growth inhibition with respect to the control. Then, diatom cultures were centrifuged at 3,000 *g *for 10 min. The supernatant was removed and the cell pellet resuspended in 0.5 mL PBS buffer, on ice (Gibco, Invitrogen, Paisley, UK). Protease inhibitors (cocktail for plant cell and tissue extracts, Sigma) were included when cells were harvested for protein extraction. The suspension was transferred to 1.5 mL tubes and centrifuged at 10,000 *g *for 10 min. Then, supernatants were removed and the remaining cell pellets were frozen at -80°C. A total of three biological replicates were performed for the BaP-exposed and control conditions in consecutive weeks.

### Total protein extraction and iTRAQ labeling

For protein extraction, the frozen cell pellet was allowed to thaw on ice, and resuspended with 200 μL of buffer containing 50 mM MOPS, 0.05% Triton X-100, 10 mM DTT and protease inhibitor cocktail (Sigma). Cell lysis was performed using homogenization with a IKA^® ^T10 basic ULTRA-TURRAX, for periods of 2 min on ice, briefly spinning down to remove any foam created by the procedure. Around 5-7 cycles homogenization were necessary for complete homogenization, which was verified by inspection on a microscope. Finally, the samples were sonicated for 30 min on ice and the lysate was centrifuged at 16,000 *g *for 10 min. Then, the supernatant was transferred into a clean microcentrifuge tube. Acetone precipitation of the proteins was usually required to concentrate the protein extract and done systematically. This was achieved by the addition of 6 volumes of cold acetone to the protein samples, mixing by gently vortexing and incubating at -20°C overnight. After centrifugation at 16,000 *g *for 10 min and removal of the supernatant, the pellet was dissolved in 50 μL TEAB buffer containing 0.1% Triton X-100. Several steps of sonication on ice and vortexing were required for complete solubilization. Sample reduction with TCEP was performed according to the iTRAQ protocol (Applied Biosystems) for 1 h at 60°C. Any insoluble material was removed by centrifugation. Protein concentration was measured using a colorimetric method (Bradford reagent, Bio-Rad), according to the manufactors protocol. A total amount of 60 μg from each sample was transferred into new tubes and adjusted to the same volume with TEAB buffer. Cysteine blocking with methyl methanethiosulfonate (MMTS) for 10 min was performed after which trypsin (Proteomics grade, Sigma) was added at a trypsin:protein ratio of 1:20 and digestion occurred for 16 h at 37°C. Protein samples before and after digestion were taken and run on SDS-PAGE both to confirm similar amounts of protein extract in each sample as well as to confirm complete protein digestion after the incubation with trypsin. Protein visualization was done by silver staining with the SilverQuest Silver Staining Kit (Invitrogen) following the standard protocol provided by the company.

Each iTRAQ 8-plex reagent (Applied Biosystems) was dissolved in 50 μL isopropanol and added to a total amount of 53 μg of peptide sample. Verification of the samples pH (7.5-8.5) was done and labeling occurred for 2 h at room temperature. Samples were frozen at -80°C and shipped within a day in dry ice to Proteome Factory, Germany for SCX separation and MS/MS analysis.

### Peptide SCX separation

Efficiency of labeling from each sample was verified by mass spectrometry before the eight samples were pooled. SCX separation used an Agilent 1100 HPLC system with a PolySULFOETHYL A column (200 mm × 2.1 mm, 5 μm, 200 Å, PolyLC, USA). The iTRAQ labeled sample was dried by speedvac and resuspended in 1.5 mL 0.1% formic acid/25% acetonitrile and the pH adjusted to about pH 2.5. The following separation gradient with buffer A (0.1% formic acid/25% acetonitrile) and buffer B (500 mM KCl/0.1% formic acid/25% acetonitrile) was used at flow rate 200 μl/min (0%B for 10 min; 40%B for 45 min; 70%B for 57.5 min and 100%B for 65 min). 18 SCX fractions were collected in a microtiter plate and dried by Speedvac for subsequent nanoLC-ESI-QStar MS.

### nanoLC-QStar MS analysis for protein identification and quantification

Protein identification and quantification of iTRAQ reporter ions were performed using nanoLC-ESI-MS/MS. The MS system consisted of an Agilent 1100 nanoLC system (Agilent, Germany), PicoTip emitter (New Objective, USA) and a Qstar XL MS (ABI, Foster City, USA). Dried SCX peptide fractions were resuspended in 80 μl 0.1% formic acid/1% acetonitrile in MilliQ water. After trapping 40 μl sample and desalting the peptides on enrichment column (Zorbax SB C18, 0.3 mm × 5 mm, Agilent) using 1% acetonitrile, 0.1% formic acid solution for 10 minutes, peptides were separated on Zorbax 300 SB C18, 75 μm × 150 mm column (Agilent, Germany) using an acetonitrile, 0.1% formic acid gradient from 5% to 35% acetonitrile within 110 minutes. MS spectra were automatically taken by Qstar XL according to manufacturer's instrument settings for nanoLS-ESI-MS/MS analyses. Proteins were identified and quantified using MS/MS ion search of Mascot 2.2.06 search engine (Matrix Science, London) with iTRAQ-8plex quantification, using a peptide mass tolerance of 150 ppm, a fragment mass tolerance of 0.4 Da and two missed cleavages. A parallel database search was done using the Paragon algorithm as implemented in Protein Pilot 3.0 software (Applied Biosystems), using the customized search settings. Searches were performed against a protein database containing the entire proteome of *Thalassiosira pseudonana*, (downloaded from UniProtKB/Swiss-Prot), with 11842 sequences [8,36], concatenated with a reversed "decoy" version to calculate the false discovery rate, and in addition, a search on the entire NCBI database was performed to exclude the presence of common protein contaminants and confirm the *Thalassiosira pseudonana *origin of the protein hits. For both analyses, the relative quantitative protein ratios relative to iTRAQ reporter ion 113 were obtained, and median ratio normalization was performed, as recommended for complex protein samples. Relative quantitative protein ratios compared to the iTRAQ reporter ion 113 were imported to an Excel (Microsoft, Redmond, WA, USA) spreadsheet and a 2-tailed, unpaired statistical evaluation was run to assess the significance in the observed differences in iTRAQ reporter quantifications between the three replicates of the two sample groups. All identified proteins were verified in Swiss-Prot and JGI http://genome.jgi-psf.org/Thaps3/Thaps3.home.html databases for the annotation (Additional file 3: Table S1).

### RNA isolation and qRT-PCR

Extraction of total RNA from three biological replicates in both control and BaP-exposed conditions, was performed using the Trizol LS (Invitrogen) protocol, as described previously [11], using 20-40 million cells per extraction. For the reverse transcription, the RNA was first treated with DNase I (Roche Diagnostics, Basel, Switzerland), according to the manufacturer's protocol, and then transcribed to cDNA using oligodT primers and SuperScript II Reverse Transcriptase Kit as recommended (Invitrogen). The purity and integrity of the RNA was verified by absorbance measurements at 260 nm with a Nanodrop ND-1000 spectrophotometer (NanoDrop Technologies Inc, USA) and by electrophoretic separation with a Bioanalyzer using the RNA Nano kit (Agilent Technologies).

Regulation of the expression of the gene *sit1*, coding for silicon transporter 1 (Swiss-Prot:Q0QVM8) was analyzed by qRT-PCR, using the forward primer with sequence 5'TTGCCGAGGATGCCTAAACTT3', the reverse primer with sequence 5'TGACGAGCTACTGCAGGTTCA3' and the TaqMAN MGB 6-FAM probe 5'TGGCAATCTGTTGTAAAT3'.

Amplification reactions were performed using the Master Mix 2× (Applied Biosystems), 0.9 mM of reverse and forward primers, 0.1 mM probe, and 2.5 μL of cDNA in a final volume of 25 μL. Samples were loaded in triplicate on 96-well optical reaction plates and qRT-PCR was performed in the ABI Prism 7900HT sequence detection system (Applied Biosystems). Each cycle of the thermal amplification followed the universal protocol according to the manufacturer, i.e. 2 min preheating at 50°C, 10 min at 95°C, followed by 40 cycles with 15 s at 95°C and 1 min at 60°C. Gene expression data from qRT-PCR were evaluated using Q-Gene [37], which takes into account the amplification efficiencies of target and reference genes. For all qRT-PCR results, glyceraldehyde-3-phosphate dehydrogenase (*gapdh*) was used as housekeeping gene, for which sequences of the specific primers have been previously published [11].

## Abbreviations

BaP: Benzo(a)pyrene; iTRAQ: 8-plex amine-modifying labeling reagents for multiplexed relative and absolute protein quantitation; DTT: dithiotreitol; ESI: electrospray ionization; GAPDH: glyceraldehydes-3-phosphate dehydrogenase; iTRAQ: isobaric tag for relative and absolute quantitation; LC: liquid chromatography; PAHs: polycyclic aromatic hydrocarbons; PCA: principle component analysis; qRT-PCR: quantitative real-time polymerase chain reaction; SCX: strong cation exchange; SIT1: silicon transporter 1; MS/MS: tandem mass spectrometry; TEAB: triethylammonium bicarbonate; TCEP: tris-(2-carboxyethyl)-phosphine.

## Authors' contributions

RNC participated in the design of the study, drafted the manuscript and carried out all the experiments and data analysis described except for the SCX separation, the nanoLC-QStar MS analysis and the database search, which have been performed as a service at the Proteome Factory (Germany) who also provided a description for the method. TL participated in the design of the study, designed the primers for qRT-PCR and assisted in drafting the manuscript. All authors read and approved the final manuscript.

## Supplementary Material

Additional file 1 **Workflow for the identification of protein changes induced by BaP treatment in *T. pseudonana***. Diatom cells were inoculated into fresh medium, cultured for 24 h and exposed to BaP (36 μg/L) or just the solvent (control) for 24 h, in three biological replicates. Following cell lysis, 60 μg of protein were alkylated, reduced and digested with trypsin. Peptides were then labeled with iTRAQ reagents and pooled as shown. Strong cation exchange was then used to remove free iTRAQ reagent and to fractionate peptides for subsequent separation and peptide analysis by LC-MS/MS. MS/MS data was analyzed using Mascot and ProteinPilot software. Click here for file

Additional file 2 **Silver staining of protein extract and tryptic digest run on SDS-PAGE**. The figure shows the similar amount of proteins run for three biological replicates. Control protein extracts, labeled with iTRAQ 113, 115 and 119 were loaded onto lanes 1, 5 and 9, respectively, while BaP-exposed protein extracts, labeled with iTRAQ 114, 116 and 121 were loaded onto lanes 3, 7 and 11, respectively. Tryptic digested products were loaded on the neighboring lanes to the right of the corresponding protein extracts, and show the protein digestion was complete under the used conditions. Molecular weight markers were loaded onto lanes labeled with M. Click here for file

Additional file 3 **Protein identification in *T. Pseudonana***. The file shows Table S1 listing all the proteins identified by MS/MS from the *T. pseudonana *extract with a minimum of 2 peptides at >95% confidence. Click here for file

Additional file 4 **Peptide fragmentation spectra used for the identification and relative quantification of proteins that have been included in **Table 1**as potentially regulated**. The file contains the fragmentation spectra of the peptide fragment identified with high confidence (> 95%) as well as the fragmentation spectra of other peptides identified with lower confidence, for six different proteins (Supplemental Figures S1-S6). The reporter ion signals shown in the low-mass region of the spectra were used to determine the relative amount of the protein in *T. pseudonana *exposed to BaP (iTRAQ 114, 116 and 121) when compared to control conditions (iTRAQ 113, 115 and 119). Click here for file

Additional file 5 **Principle component analysis for the quantification of the iTRAQ labels in the different experiments**. The analysis shows a clustering of the proteome analysis from control diatom cultures, labeled with iTRAQ 113, 115 and 119, as well as a clustering of the proteome analysis from the BaP-exposed conditions, labeled with iTRAQ 114, 116 and 121. An additional experiment has been incorporated in the MS/MS analysis, consisting of control and BaP-exposed conditions, labeled with iTRAQ 117 and 118, respectively, but were not considered for the overall quantification analysis. In this experiment, the same starting density of 0.5 million cells/mL was used, but from a culture in the resting phase. We observed that the growth in the diatom cultures was largely reduced from the expected values, and was also observed after the addition of solvent or BaP. We decided to use the cells arising from this experiment with the remaining iTRAQ labels from the 8-plex kit, to see whether under these abnormal cell culture circumstances, we could still observe significant differences between control and exposure conditions. It is evident that both conditions in this experiment sit as outliers on the graph and in addition, they seem to be clustered together. In fact, from the quantification analysis, it was observed that both 117 and 118 labels showed rather consistent values for most of the peptides analyzed, and not in agreement with the experimental conditions, i.e. control or BaP-exposed. It is interesting to note that the reduced cell growth in this culture, greatly affected the outcome of the quantification. Thus, the apparent sensitivity of these cells seemed to be more relevant than the exposure conditions themselves. These findings, although not unexpected, truly support the importance of reproducibility in biomarker research, since any differences in the initial state of the cells has a substantial effect on the biomolecular pool and thus masks any potential differences of expression, either at gene or protein level. Click here for file
