# Supplementary material for: Proteomic analysis of the marine diatom Thalassiosira pseudonana upon exposure to benzo(a)pyrene
Source: BMC Genomics. 2011 Mar 24;12:159. doi: 10.1186/1471-2164-12-159 (PMC3076255; doi:10.1186/1471-2164-12-159)
Supplement: Additional file 1 — Workflow for the identification of protein changes induced by BaP treatment in T. pseudonana. Diatom cells were inoculated into fresh medium, cultured for 24 h and exposed to BaP (36 μg/L) or just the solvent (control) for 24 h, in three biological replicates. Following cell lysis, 60 μg of protein were alkylated, reduced and digested with trypsin. Peptides were then labeled with iTRAQ reagents and pooled as shown. Strong cation exchange was then used to remove free iTRAQ reagent and to fractionate peptides for subsequent separation and peptide analysis by LC-MS/MS. MS/MS data was analyzed using Mascot and ProteinPilot software. [file 1471-2164-12-159-S1.PPT]

## Slide 1
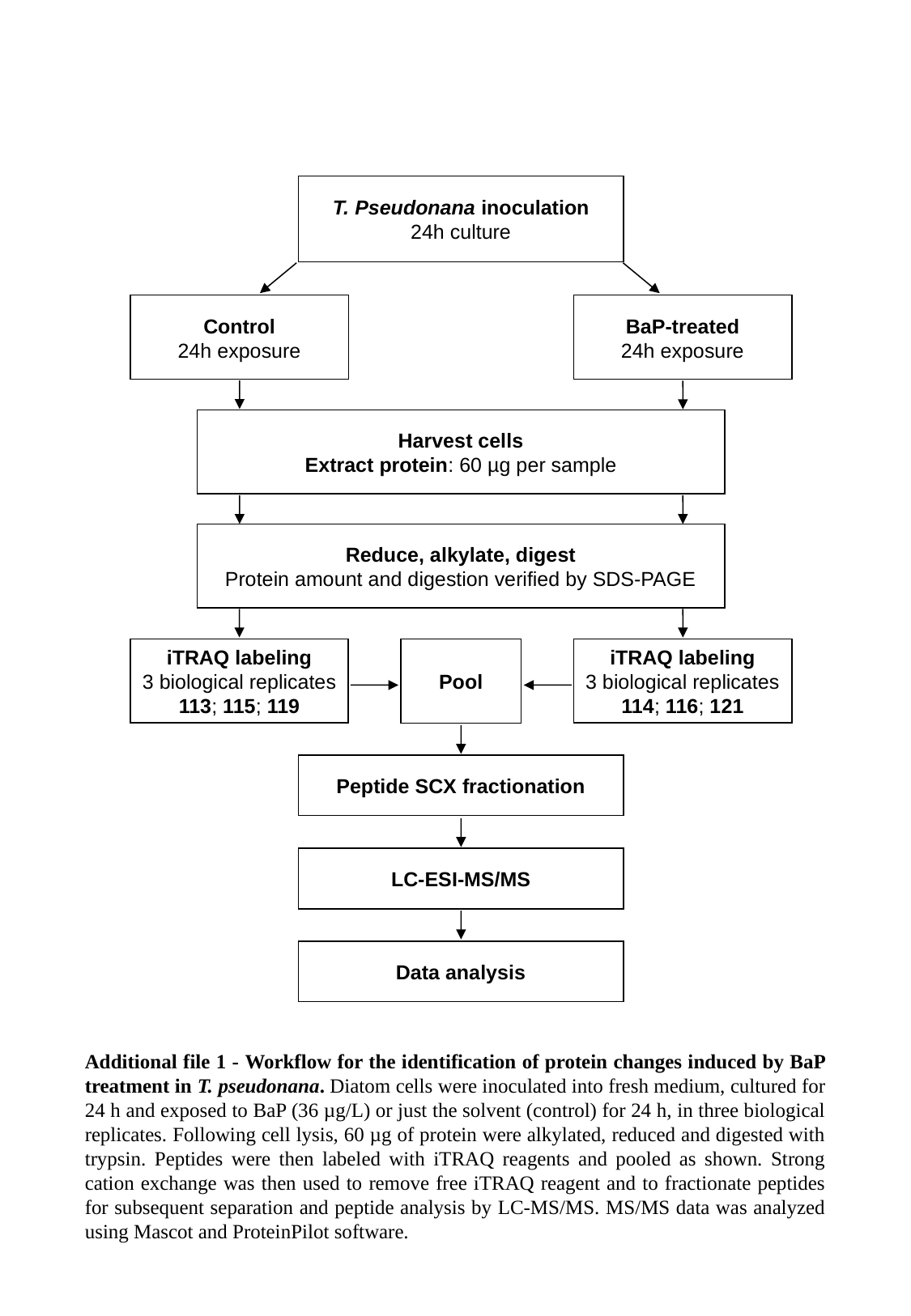

T. Pseudonana inoculation
24h culture
Control
24h exposure
BaP-treated
24h exposure
Harvest cells
Extract protein: 60 µg per sample
Reduce, alkylate, digest
Protein amount and digestion verified by SDS-PAGE
iTRAQ labeling
3 biological replicates
113; 115; 119
iTRAQ labeling
3 biological replicates
114; 116; 121
Pool
Peptide SCX fractionation
LC-ESI-MS/MS
Data analysis
Additional file 1 - Workflow for the identification of protein changes induced by BaP treatment in T. pseudonana. Diatom cells were inoculated into fresh medium, cultured for 24 h and exposed to BaP (36 µg/L) or just the solvent (control) for 24 h, in three biological replicates. Following cell lysis, 60 µg of protein were alkylated, reduced and digested with trypsin. Peptides were then labeled with iTRAQ reagents and pooled as shown. Strong cation exchange was then used to remove free iTRAQ reagent and to fractionate peptides for subsequent separation and peptide analysis by LC-MS/MS. MS/MS data was analyzed using Mascot and ProteinPilot software.
