# Supplementary material for: Proteomic analysis of the marine diatom Thalassiosira pseudonana upon exposure to benzo(a)pyrene
Source: BMC Genomics. 2011 Mar 24;12:159. doi: 10.1186/1471-2164-12-159 (PMC3076255; doi:10.1186/1471-2164-12-159)
Supplement: Additional file 2 — Silver staining of protein extract and tryptic digest run on SDS-PAGE. The figure shows the similar amount of proteins run for three biological replicates. Control protein extracts, labeled with iTRAQ 113, 115 and 119 were loaded onto lanes 1, 5 and 9, respectively, while BaP-exposed protein extracts, labeled with iTRAQ 114, 116 and 121 were loaded onto lanes 3, 7 and 11, respectively. Tryptic digested products were loaded on the neighboring lanes to the right of the corresponding protein extracts, and show the protein digestion was complete under the used conditions. Molecular weight markers were loaded onto lanes labeled with M. [file 1471-2164-12-159-S2.PPT]

## Slide 1
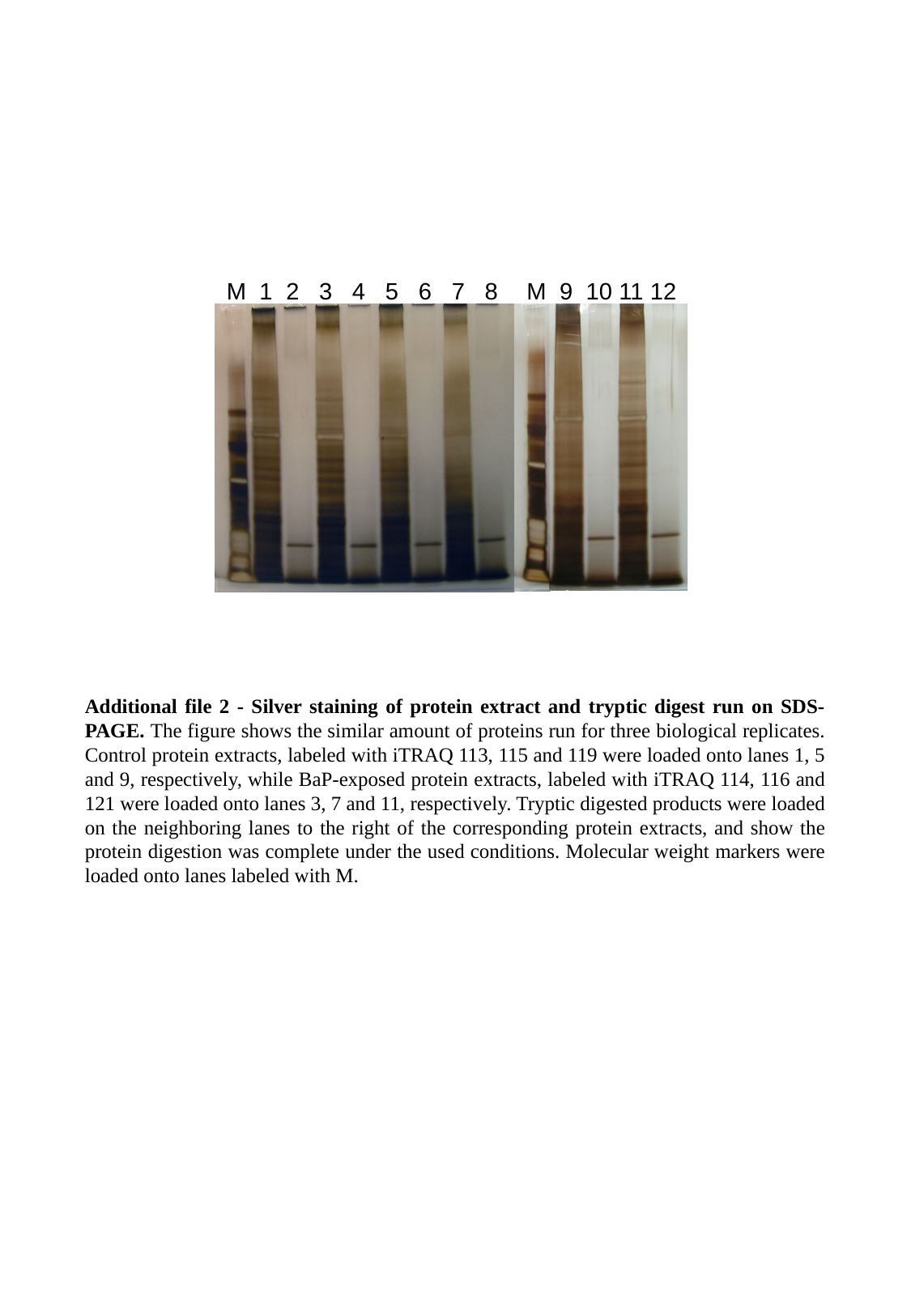

M 1 2 3 4 5 6 7 8
M 9 10 11 12
Additional file 2 - Silver staining of protein extract and tryptic digest run on SDS-PAGE. The figure shows the similar amount of proteins run for three biological replicates. Control protein extracts, labeled with iTRAQ 113, 115 and 119 were loaded onto lanes 1, 5 and 9, respectively, while BaP-exposed protein extracts, labeled with iTRAQ 114, 116 and 121 were loaded onto lanes 3, 7 and 11, respectively. Tryptic digested products were loaded on the neighboring lanes to the right of the corresponding protein extracts, and show the protein digestion was complete under the used conditions. Molecular weight markers were loaded onto lanes labeled with M.
