# Supplementary material for: Proteomic analysis of the marine diatom Thalassiosira pseudonana upon exposure to benzo(a)pyrene
Source: BMC Genomics. 2011 Mar 24;12:159. doi: 10.1186/1471-2164-12-159 (PMC3076255; doi:10.1186/1471-2164-12-159)
Supplement: Additional file 3 — Protein identification in T. Pseudonana. The file shows Table S1 listing all the proteins identified by MS/MS from the T. pseudonana extract with a minimum of 2 peptides at >95% confidence. [file 1471-2164-12-159-S3.DOC]

| **Table S1 -** List of proteins identified with by MS/MS from the *T. pseudonana* extract with a minimum of 2 peptides at >95% confidence   | **Protein information^a^** | | | | | | **Protein Identification^b^** | | | | | --- | --- | --- | --- | --- | --- | --- | --- | --- | --- | | **Protein name** | **Accession** | **Gene** | **MW (kDa)** | **pI** | **GO** | **Peptides** | **Total** | **%Cov**  **(50)** | **%Cov**  **(95)** | | ATP synthase subunit beta | B8LET6 | THAPSDRAFT_bd1563 | 51.1 | 4.7 | ATP synthesis | 39 | 43.9 | 62.0 | 59.9 | | Ribulose-bisphosphate carboxylase, RuBisCo | B8LER9 | RBL | 54.3 | 6.1 | carbon fixation | 36 | 34.9 | 48.0 | 40.6 | | ATP synthase subunit alpha | B8LER1 | atpA | 54.0 | 4.9 | ATP synthesis | 28 | 38.4 | 47.5 | 46.1 | | Putative uncharacterized protein | B8C7S8 | THAPSDRAFT_7881 | 31.2 | 4.9 | glycolysis | 22 | 29.3 | 73.1 | 73.1 | | Phosphoglycerate kinase | B5YN92 | THAPS_270304 | 46.4 | 4.9 | glycolysis | 19 | 30.7 | 41.9 | 39.7 | | Elongation factor Tu | A0T100 | tufA | 44.5 | 4.8 | protein biosynthesis | 19 | 23.5 | 50.1 | 50.1 | | Predicted protein | B8C7S9 | THAPSDRAFT_23918 | 31.2 | 4.7 |  | 19 | 27.3 | 67.6 | 67.6 | | Transketolase | B8BTR4 | TKT2 | 71.7 | 5.0 | metabolic process | 17 | 26.9 | 36.2 | 29.1 | | Putative uncharacterized protein | B8CGK1 | GLNN | 69.2 | 5.1 | metabolic process | 12 | 24.5 | 39.4 | 21.8 | | Putative uncharacterized protein | B8LE18 | THAPSDRAFT_bd1766 | 45.6 | 4.8 |  | 12 | 10.0 | 14.4 | 14.4 | | Glyceraldehyde-3-phosphate dehydrogenase GADP1 | B8BQU2 | GAPD1 | 39.6 | 5.2 | oxidoreductase | 11 | 20.1 | 38.7 | 33.9 | | RL4e, ribosomal protein 4e 60S | B8C239 | RL4e | 41.0 | 10.3 | translation | 11 | 18.7 | 37.0 | 32.0 | | Ribulose-1,5-bisphosphate carboxylase/oxygenase | A0T0N5 | rbcS | 15.8 | 5.0 | carbon fixation | 10 | 17.1 | 79.9 | 69.1 | | Ascorbate peroxidase | B8C590 | ApxS | 32.2 | 4.8 | oxidation reduction | 9 | 14.4 | 34.7 | 34.7 | | Predicted protein | B8BVI4 | THAPSDRAFT_2848 | 15.5 | 9.2 | photosynthesis | 9 | 10.9 | 51.3 | 46.6 | | Fucoxanthin chlorophyll a/c protein 8 | B8C261 | Lhcf8 | 27.8 | 5.1 | photosynthesis | 9 | 9.7 | 28.4 | 28.4 | | Putative uncharacterized protein | B8C8L8 | THAPSDRAFT_24060 | 48.2 | 4.9 | metabolic process | 9 | 8.1 | 15.5 | 10.0 | | Putative uncharacterized protein | B8C8L9 | THAPSDRAFT_269459 | 42.8 | 5.0 | metabolic process | 9 | 6.4 | 11.9 | 11.9 | | Acetyl-coa carboxylase | B5YMF5 | ACC | 228.3 | 5.0 | fatty acid biosynthesis | 8 | 19.9 | 9.9 | 5.2 | | ATP synthase gamma chain | B8C0W4 | THAPSDRAFT_40156 | 39.9 | 6.7 | ATP synthesis | 8 | 14.2 | 30.3 | 30.3 | | Putative uncharacterized protein | B8C6L5 | THAPSDRAFT_35934 | 13.5 | 4.9 | electron transport | 8 | 11.4 | 48.9 | 48.9 | | Phosphoadenosine-phosphosulphate reductase | B8CCL3 | THAPSDRAFT_24887 | 49.0 | 5.0 | cell redox homeostasis | 7 | 15.7 | 30.8 | 21.9 | | Mitochondrial chaperonin | B5YLQ5 | HSP60 | 58.5 | 4.8 | protein folding | 7 | 14.8 | 21.5 | 17.4 | | Phosphoenolpyruvate carboxylase | B8BYW8 | PPC1 | 97.9 | 5.5 | tricarboxylic acid cycle | 7 | 14.2 | 10.9 | 9.6 | | Citrate synthase | B8CEG2 | CSN1 | 52.3 | 6.2 | tricarboxylic acid cycle | 7 | 14.0 | 19.2 | 17.1 | | Ferredoxin-NADP reductase | B8CGL9 | THAPSDRAFT_25892 | 37.8 | 5.7 | oxidation reduction | 7 | 11.7 | 23.6 | 18.3 | | Predicted protein | B8C486 | THAPSDRAFT_23228 | 58.2 | 5.1 | oxidation reduction | 7 | 10.3 | 14.6 | 14.6 | | Triosephosphate isomerase | B8C246 | THAPSDRAFT_28239 | 65.3 | 5.2 | oxidation reduction | 7 | 11.5 | 16.1 | 13.8 | | Predicted protein | B8CAG8 | THAPSDRAFT_24512 | 26.8 | 5.2 |  | 7 | 10.2 | 33.1 | 33.1 | | S-adenosylmethionine synthetase | B8BY55 | AMS1 | 50.4 | 5.2 | metabolic process | 6 | 14.0 | 27.5 | 16.3 | | Glycine decarboxylase p-protein | B8BX31 | GDCP | 104.7 | 5.3 | oxidation reduction | 6 | 13.7 | 13.4 | 9.9 | | Predicted protein | B8C0L7 | THAPSDRAFT_270396 | 42.8 | 4.9 | glycolysis | 6 | 12.0 | 23.5 | 21.0 | | Predicted protein | B8C0D4 | THAPSDRAFT_28049 | 29.2 | 9.2 | translation | 6 | 11.8 | 27.4 | 27.4 | | Oxygen-evolving enhancer protein 1 | B8C4I5 | PsbO | 32.1 | 5.3 | photosynthesis | 6 | 11.0 | 34.7 | 23.3 | | RS19, ribosomal protein 19 40S | B8C3T8 | RS19 | 16.7 | 8.7 | translation | 6 | 10.0 | 53.4 | 35.8 | | Phosphoribulokinase | B8BZ40 | PRK1 | 42.4 | 4.9 | metabolic process | 6 | 8.8 | 12.6 | 12.6 | | RL13A, ribosomal protein 13A | B8C0R3 | RL13A | 22.6 | 10.4 | translation | 6 | 7.4 | 22.4 | 18.9 | | Predicted protein | B8CG97 | THAPSDRAFT_25840 | 25.3 | 5.2 |  | 6 | 7.1 | 21.9 | 15.6 | | CPSase | B8BZG0 | THAPSDRAFT_40323 | 163.6 | 5.3 | metabolic process | 5 | 11.1 | 7.2 | 3.5 | | 40S ribosomal protein-like protein | B8BYG9 | RSP1 | 27.3 | 5.7 | translation | 5 | 11.1 | 36.6 | 23.9 | | Phospoenolpyruvate carboxylase | B8C1R7 | PPC2 | 95.4 | 6.4 | tricarboxylic acid cycle | 5 | 10.3 | 12.5 | 8.9 | | Predicted protein | B8LER0 | THAPSDRAFT_bd2073 | 56.4 | 6.4 | photosynthesis | 5 | 9.5 | 12.0 | 9.4 | | RL5, ribosomal protein 5 | B5YLN7 | RL5 | 35.3 | 8.5 | translation | 5 | 9.1 | 21.9 | 21.9 | | Predicted protein | B8C4T7 | THAPSDRAFT_6610 | 63.0 | 4.8 | metabolic process | 5 | 8.9 | 12.4 | 12.4 | | Predicted protein | B8CGI1 | THAPSDRAFT_38807 | 16.6 | 4.5 | metabolic process | 5 | 8.0 | 49.7 | 49.7 | | Predicted protein | B8LEA0 | THAPSDRAFT_bd1820 | 26.5 | 5.0 |  | 5 | 6.7 | 20.8 | 20.8 | | Cytochrome c550, PsbV | B8LES1 | PsbV | 17.8 | 6.8 | photosynthesis | 5 | 6.6 | 23.3 | 18.4 | | Predicted protein | B8LEJ5 | THAPSDRAFT_bd1863 | 20.5 | 5.9 |  | 5 | 5.6 | 19.2 | 15.7 | | RS24, ribosomal protein 24 | B8C5V1 | RS24 | 15.3 | 10.5 | translation | 5 | 5.1 | 22.6 | 16.8 | | Fucoxanthin-chlorophyll a-c binding protein | B8BVI1 | Lhcf11 | 21.0 | 4.7 | photosynthesis | 5 | 4.0 | 13.8 | 13.8 | | Heat shock protein/chaperone | B8BWB9 | hsp70_4 | 70.5 | 4.7 | stress response | 4 | 10.5 | 14.3 | 8.5 | | Predicted protein | B5YM44 | THAPS_269961 | 28.9 | 10.4 |  | 4 | 9.7 | 27.8 | 20.9 | | Putative uncharacterized protein | B8C2R7 | THAPSDRAFT_262506 | 48.3 | 4.3 |  | 4 | 9.6 | 17.1 | 12.0 | | Porphobilinogen deaminase | B8CA57 | PbgD | 35.9 | 4.7 | tetrapyrrole biosynthesis | 4 | 9.5 | 26.1 | 19.4 | | RL10, ribosomal protein 10 | B8C3Y3 | RL10 | 24.7 | 10.1 | translation | 4 | 8.7 | 25.8 | 19.8 | | NADPH nitrite reductase | B8BTJ8 | NIR_1 | 109.7 | 5.0 | oxidation-reduction | 4 | 8.3 | 4.2 | 4.2 | | Enolase | B8C355 | ENO2 | 46.7 | 4.9 | glycolysis | 4 | 8.2 | 16.1 | 16.1 | | RS6, ribosomal protein 6 | B8CCL8 | RS6 | 27.6 | 10.9 | translation | 4 | 8.2 | 21.4 | 18.5 | | RL3, ribosomal protein 3 | B5YMS4 | RL3 | 44.6 | 10.2 | translation | 4 | 8.1 | 16.7 | 10.9 | | Predicted protein | B8BVP1 | THAPSDRAFT_21517 | 58.9 | 6.7 | metabolic process | 4 | 8.0 | 7.2 | 7.2 | | 60S ribosomal protein L6 | B8CBR7 | RL6 | 19.5 | 9.8 | translation | 4 | 8.0 | 29.2 | 29.2 | | Aspartate aminotransferase | B8BT02 | THAPSDRAFT_31394 | 44.3 | 4.7 | biosynthetic process | 4 | 7.9 | 16.1 | 14.4 | | Predicted protein | B8LCK9 | THAPSDRAFT_10417 | 22.9 | 4.9 |  | 4 | 7.5 | 25.6 | 21.5 | | Predicted protein | B8LE17 | THAPSDRAFT_bd1765 | 32.1 | 5.4 |  | 4 | 7.5 | 18.2 | 18.2 | | Predicted protein | B8BU42 | THAPSDRAFT_21235 | 41.9 | 5.6 | RNA binding | 4 | 7.4 | 12.1 | 12.1 | | Putative uncharacterized protein | B8CET1 | THAPSDRAFT_25629 | 86.4 | 4.9 | translational elongation | 4 | 7.4 | 8.7 | 8.7 | | Predicted protein | B8LCF3 | THAPSDRAFT_37809 | 14.1 | 9.9 | translation | 4 | 7.0 | 41.5 | 41.5 | | Fucoxanthin chlorophyll a/c protein 6 | B8BX92 | Lhcf6 | 21.8 | 5.2 | photosynthesis | 4 | 6.9 | 24.4 | 18.9 | | Putative uncharacterized protein | B8BT16 | THAPSDRAFT_997 | 38.3 | 5.4 | metabolic process | 4 | 6.9 | 19.0 | 15.1 | | Predicted protein | B8C272 | THAPSDRAFT_40669 | 17.6 | 9.8 | translation | 4 | 6.7 | 24.8 | 24.8 | | Predicted protein | B8BQT6 | THAPSDRAFT_20812 | 16.2 | 5.3 |  | 4 | 6.4 | 35.1 | 28.4 | | RS20, ribosomal protein 20 | B8C138 | RS20 | 13.7 | 9.8 | translation | 4 | 6.3 | 27.4 | 27.4 | | Ribosomal protein S8 | B8CB96 | RS8 | 22.6 | 10.4 | translation | 4 | 6.3 | 26.5 | 20.5 | | Ascorbate peroxidase | B8CFA9 | THAPSDRAFT_38724 | 29.3 | 5.2 | oxidation reduction | 4 | 6.0 | 12.6 | 12.6 | | Glyceraldehyde-3-phosphate dehydrogenase GAPD4 | B8C303 | GAPD4 | 36.6 | 5.9 | metabolic process | 4 | 5.9 | 16.0 | 14.0 | | Fucoxanthin chlorophyll a/c light-harvesting protein | B8BX93 | Lhcf7 | 20.9 | 4.7 | photosynthesis | 4 | 4.0 | 13.9 | 13.9 | | Phosphoglycerate kinase | B8LCH6 | PGK3 | 43.0 | 5.0 | glycolysis | 4 | 7.2 | 9.4 | 6.9 | | Chaperone protein dnaK | A0T0X1 | dnaK | 65.3 | 4.8 | protein folding | 3 | 9.8 | 15.6 | 7.3 | | Adenosylhomocysteinase | B8C553 | AHC3 | 52.3 | 5.1 | metabolic process | 3 | 7.9 | 11.6 | 7.3 | | 3-isopropylmalate dehydrogenase | B8C2I2 | THAPSDRAFT_268875 | 44.3 | 4.7 | oxidation reduction | 3 | 7.4 | 14.2 | 7.7 | | Heat shock protein 70 | B8C635 | hsp70 | 71.2 | 4.8 | stress response | 3 | 7.3 | 7.8 | 6.3 | | Predicted protein | B8BSZ5 | THAPSDRAFT_976 | 17.2 | 4.4 |  | 3 | 7.1 | 22.0 | 22.0 | | Putative uncharacterized protein | B8C9U7 | THAPSDRAFT_29506 | 11.4 | 6.6 | protein folding | 3 | 7.1 | 49.5 | 39.1 | | Predicted protein | B8BZI8 | THAPSDRAFT_270365 | 76.2 | 6.2 | oxidation reduction | 3 | 7.1 | 7.3 | 5.9 | | Pyruvate carboxylase-like protein | B8CE42 | THAPSDRAFT_269908 | 126.2 | 5.2 | gluconeogenesis | 3 | 6.9 | 5.7 | 3.6 | | N-acetylornithine aminotransferase | B8CGH5 | THAPSDRAFT_270136 | 50.7 | 5.6 | metabolic process | 3 | 6.9 | 11.7 | 8.6 | | Fucoxanthin chlorophyll a/c protein 2 | B8CEV8 | Lhcf2 | 21.1 | 4.5 | photosynthesis | 3 | 6.9 | 27.3 | 27.3 | | RS5, ribosomal protein 5 | B8CC69 | RS5 | 24.4 | 6.5 | translation | 3 | 6.8 | 19.3 | 13.3 | | RL18, ribosomal protein 18 | B5YMU6 | RL18 | 21.3 | 11.0 | translation | 3 | 6.7 | 27.6 | 23.4 | | 14-3-3-like protein | B8BX06 | THAPSDRAFT_26146 | 27.9 | 4.6 | protein binding | 3 | 6.6 | 12.5 | 12.5 | | Guanine nucleotide binding protein beta subunit-like protein | B8BQ72 | THAPSDRAFT_26063 | 36.0 | 6.1 |  | 3 | 6.5 | 21.8 | 19.3 | | RS9, ribosomal protein 9 | B8C1H2 | RS9 | 21.8 | 10.1 | translation | 3 | 6.5 | 26.8 | 22.6 | | Chaperone, heat shock protein 70 | B5YMV8 | dnaK | 72.2 | 4.9 | protein folding/ stress response | 3 | 6.5 | 9.1 | 6.0 | | RL12, ribosomal protein 11 | B8BTS3 | RL12 | 17.4 | 8.7 | translation | 3 | 6.3 | 35.4 | 29.9 | | RL18a, ribosomal protein 18a | B8BZG7 | RL18a | 21.1 | 10.4 | translation | 3 | 6.2 | 15.9 | 15.9 | | Aconitase hydratase 2 | B8C3V9 | acnB | 94.4 | 4.8 | tricarboxylic acid cycle | 3 | 6.1 | 6.9 | 4.7 | | RS13, ribosomal protein 13 | B8C0K5 | RS13 | 17.1 | 10.3 | translation | 3 | 6.1 | 23.2 | 18.5 | | RS4, ribosomal protein 4 | B8BVV7 | RS4 | 29.3 | 9.8 | translation | 3 | 6.1 | 11.5 | 11.5 | | Predicted protein | B8LES9 | THAPSDRAFT_bd1339 | 15.5 | 9.4 | photosynthesis | 3 | 6.1 | 36.7 | 30.9 | | Predicted protein | B8BQS1 | THAPSDRAFT_20804 | 20.7 | 4.5 |  | 3 | 6.0 | 25.4 | 15.7 | | Predicted protein | B8BRY1 | THAPSDRAFT_1786 | 96.5 | 4.4 |  | 3 | 6.0 | 6.7 | 6.7 | | Predicted protein | B8C770 | THAPSDRAFT_23808 | 21.1 | 4.8 | photosynthesis | 3 | 6.0 | 22.7 | 22.7 | | Predicted protein | B8CCU1 | THAPSDRAFT_24932 | 23.9 | 4.8 | oxidation reduction | 3 | 5.8 | 26.9 | 19.4 | | Predicted protein | B8LER4 | THAPSDRAFT_bd598 | 17.4 | 4.5 | ion transport | 3 | 5.7 | 28.2 | 28.2 | | Histone H4 | B5YMD7 | H4_2 | 11.4 | 11.4 | nucleosome assembly | 3 | 5.5 | 29.1 | 29.1 | | Predicted protein | B8C0D9 | THAPSDRAFT_4820 | 45.9 | 6.4 |  | 3 | 5.5 | 12.7 | 7.1 | | Adenine nucleotide translocator | B8BR30 | ANT1 | 32.3 | 9.3 | transport | 3 | 5.4 | 14.2 | 10.3 | | Predicted protein | B8C8S6 | THAPSDRAFT_8571 | 19.1 | 5.6 |  | 3 | 5.0 | 19.7 | 15.6 | | Predicted protein | B8BYV5 | THAPSDRAFT_3817 | 49.9 | 5.0 | methylation | 3 | 4.9 | 12.2 | 6.1 | | Predicted protein | B8BSY9 | THAPSDRAFT_20603 | 22.4 | 9.0 |  | 3 | 4.9 | 24.2 | 12.3 | | Predicted protein | B8CFG4 | THAPSDRAFT_270233 | 21.8 | 4.9 |  | 3 | 5.0 | 19.3 | 13.9 | | Triose-phosphate isomerase | B5YLS7 | TPI2 | 29.0 | 4.8 | metabolic process | 3 | 4.1 | 10.9 | 10.9 | | Cold-shock DNA-binding domain-containing protein | B8C1C4 | THAPSDRAFT_40233 | 14.5 | 8.1 | transcription regulation | 3 | 4.0 | 32.4 | 32.4 | | Glyceraldehyde 3-phosphate dehydrogenase | B8C247 | gapC3 | 36.2 | 5.8 | metabolic process | 3 | 7.3 | 19.1 | 12.5 | | Enolase | B8BZT5 | ENO1 | 46.5 | 4.8 | glycolysis | 3 | 5.7 | 8.6 | 8.6 | | Peptidyl-prolyl cis-trans isomerase | B8BVR6 | PPI1 | 18.5 | 5.1 | protein folding | 3 | 3.4 | 22.5 | 22.5 | | Predicted protein | B8C7F9 | THAPSDRAFT_23855 | 10.6 | 3.8 | translation | 3 | 3.4 | 20.0 | 20.0 | | Ferredoxin | B8LES3 | petF_1 | 10.7 | 3.8 | electron transport | 3 | 2.1 | 14.1 | 14.1 | | RS15B, ribosomal protein 15B | B8BXM5 | RS15B | 16.8 | 10.3 | translation | 3 | 2.0 | 15.0 | 15.0 | | Putative uncharacterized protein | B8C8T5 | THAPSDRAFT_24248 | 167.8 | 5.6 | metabolic process | 2 | 7.7 | 6.5 | 2.0 | | RS18, ribosomal protein 18 | B8BTA9 | RS18 | 17.2 | 10.7 | translation | 2 | 6.3 | 32.9 | 14.4 | | Predicted protein | B8BSI9 | THAPSDRAFT_21081 | 53.3 | 4.9 |  | 2 | 6.2 | 9.4 | 5.3 | | Predicted protein | B8CG46 | THAPSDRAFT_25812 | 34.1 | 4.7 | translation | 2 | 6.0 | 16.4 | 8.4 | | Predicted protein | B8CDL6 | THAPSDRAFT_25042 | 63.8 | 6.3 | metabolic process | 2 | 5.6 | 8.3 | 5.3 | | RS1, ribosomal protein 1 | B8C8K9 | RS1 | 31.8 | 4.6 | translation | 2 | 5.6 | 15.0 | 8.0 | | Glycine or serine hydroxymethyltransferase | B5YLQ7 | THAPS_269942 | 58.1 | 6.6 | metabolic process | 2 | 5.5 | 9.6 | 5.5 | | 60 kDa chaperonin | A0T0X0 | groEL | 57.4 | 5.1 | protein folding | 2 | 5.5 | 9.6 | 6.2 | | Putative uncharacterized protein | B8BZE7 | THAPSDRAFT_27892 | 49.6 | 6.3 | phosphate binding | 2 | 5.3 | 9.7 | 5.7 | | Cytochrome b6-f complex iron-sulfur subunit | B8BVG6 | THAPSDRAFT_270229 | 22.2 | 5.8 | electron transport | 2 | 5.1 | 20.7 | 15.1 | | Glutamine synthetase | B8CFZ9 | GLNA | 45.6 | 5.2 | biosynthetic process | 2 | 5.1 | 12.0 | 8.2 | | RS2, ribosomal protein 2 | B8C8V8 | RS2 | 28.7 | 10.4 | translation | 2 | 5.1 | 16.4 | 9.5 | | RL7, ribosomal protein 7 | B8BV47 | RL7 | 27.1 | 10.0 | translation | 2 | 5.0 | 14.9 | 10.8 | | Superoxide dismutase | B8BXP6 | THAPSDRAFT_32874 | 27.1 | 5.4 | oxidation reduction | 2 | 5.0 | 25.9 | 14.0 | | Cysteine synthase | B8BQD6 | CYS3 | 33.5 | 5.2 | metabolic process | 2 | 4.9 | 12.9 | 9.3 | | Putative uncharacterized protein | B8CC14 | THAPSDRAFT_24769 | 18.2 | 9.7 |  | 2 | 4.9 | 22.2 | 10.8 | | Enoyl-reductase [NADH] | B8BXA1 | EAR1 | 32.8 | 5.0 | oxidation reduction | 2 | 4.9 | 20.5 | 6.1 | | 40S ribosomal protein S12 | B8CDP0 | THAPSDRAFT_37628 | 12.6 | 5.8 | translation | 2 | 4.8 | 30.4 | 21.7 | | 50S ribosomal protein L5 | A0T0Y5 | rpl5 | 27.6 | 9.6 | translation | 2 | 4.8 | 10.1 | 10.1 | | Putative uncharacterized protein | B8CDB3 | THAPSDRAFT_10234 | 47.2 | 5.8 | oxidation reduction | 2 | 4.8 | 12.2 | 5.9 | | L-lactate dehydrogenase | B8BXJ5 | THAPSDRAFT_3353 | 47.4 | 6.9 | oxidation reduction | 2 | 4.8 | 13.2 | 7.7 | | Translation factor tu domain 2 | B8C469 | THAPSDRAFT_269148 | 91.9 | 5.9 | nucleotide binding | 2 | 4.7 | 4.3 | 2.5 | | 50S ribosomal protein L11 | A0T0Q5 | rpl11 | 14.9 | 9.6 | translation | 2 | 4.7 | 33.3 | 20.6 | | Ribosomal protein L19 | B8BVM9 | RL19 | 21.3 | 11.6 | translation | 2 | 4.5 | 21.2 | 15.8 | | Dihydrolipoyl dehydrogenase | B8LC90 | DLDH2 | 52.5 | 5.3 | oxidation reduction | 2 | 4.5 | 7.4 | 5.8 | | Cysteine synthase | B8BUL0 | THAPSDRAFT_270338 | 37.9 | 5.4 | biosynthetic process | 2 | 4.5 | 10.7 | 7.4 | | Predicted protein | B8C305 | THAPSDRAFT_34104 | 13.0 | 5.0 | protein folding | 2 | 4.3 | 35.8 | 24.4 | | Fructose-bisphosphate aldolase | B8BXS1 | ALDO2 | 47.6 | 4.9 | glycolysis | 2 | 4.3 | 3.4 | 3.4 | | RS16, ribosomal protein 16 | B8BZD7 | RS16 | 16.1 | 10.1 | translation | 2 | 4.3 | 24.5 | 16.8 | | Nitrite reductase-ferredoxin dependent | B8BZ06 | NIR_2 | 59.8 | 5.5 | oxidation reduction | 2 | 4.2 | 7.1 | 4.8 | | RL8, ribosomal protein 8 | B8CCN2 | RL8 | 27.9 | 10.6 | translation | 2 | 4.2 | 14.4 | 11.7 | | Photosystem II D2 protein | A0T0T0 | psbD | 39.1 | 5.5 | photosynthesis | 2 | 4.1 | 6.3 | 6.3 | | Aspartate-ammonia ligase | B8CCA0 | ASA | 40.5 | 5.4 | biosynthetic process | 2 | 4.1 | 9.6 | 6.5 | | Psedouridylate synthase | B8CCE1 | THAPSDRAFT_269765 | 35.4 | 5.9 | RNA binding | 2 | 4.1 | 10.0 | 7.3 | | RR2, ribosomal protein 2 | B8LEQ3 | RR2 | 25.6 | 9.2 | translation | 2 | 4.1 | 15.0 | 15.0 | | RS10, ribosomal protein 10 | B8C179 | RS10 | 11.9 | 9.2 | translation | 2 | 4.1 | 23.1 | 23.1 | | Predicted protein | B8LCW8 | THAPSDRAFT_10527 | 24.0 | 4.0 | vesicle-mediated transport | 2 | 4.1 | 15.6 | 15.6 | | RL26, ribosomal protein 26 | B8C2Y2 | RL26 | 15.3 | 10.7 | translation | 2 | 4.0 | 6.6 | 6.6 | | 60S ribosomal protein L10A | B8C5C3 | RPL1 | 25.8 | 9.6 | translation | 2 | 4.0 | 10.4 | 10.4 | | Fucoxanthin chl a/c light-harvesting protein | B8C0K3 | Lhcr4 | 21.7 | 4.8 | photosynthesis | 2 | 4.0 | 18.2 | 18.2 | | Malate dehydrogenase | B8BQC2 | MDH1 | 36.7 | 6.0 | tricarboxylic acid cycle | 2 | 4.0 | 6.8 | 6.8 | | Predicted protein | B8CE88 | THAPSDRAFT_25281 | 42.1 | 6.5 | metabolic process | 2 | 4.0 | 7.5 | 7.5 | | Ribosomal protein L1 | B8LEQ4 | RK1 | 25.4 | 8.5 | translation | 2 | 4.0 | 9.1 | 9.1 | | Predicted protein | B8CA04 | THAPSDRAFT_8968 | 27.6 | 5.5 | proteasome activator | 2 | 4.0 | 10.0 | 10.0 | | 50S ribosomal protein L13 | A0T0Z5 | rpl13 | 15.6 | 9.9 | translation | 2 | 4.0 | 22.1 | 22.1 | | Predicted protein | B8CBE4 | THAPSDRAFT_29842 | 18.5 | 8.7 | oxidation reduction | 2 | 4.0 | 17.8 | 17.8 | | 30S ribosomal protein S19 | A0T0X6 | rps19 | 10.4 | 10.5 | translation | 2 | 4.0 | 28.3 | 28.3 | | RS7, ribosomal protein 7 | B8BUN1 | RS7 | 21.2 | 9.8 | translation | 2 | 4.0 | 26.6 | 26.6 | | RS15A, ribosomal protein 15 | B8C8U6 | RS15A | 14.9 | 9.8 | translation | 2 | 3.8 | 21.5 | 14.6 | | ATP synthase epsilon chain | A0T0R7 | atpE | 14.3 | 4.7 | ATP synthesis | 2 | 3.8 | 27.1 | 20.3 | | Peptidyl-prolyl cis-trans isomerase | B8C7H9 | THAPSDRAFT_29244 | 20.9 | 6.4 | protein folding | 2 | 3.7 | 11.3 | 11.3 | | Predicted protein | B8BUF2 | THAPSDRAFT_270308 | 29.1 | 5.1 | pentose-phosphate shunt | 2 | 3.7 | 10.9 | 10.9 | | Predicted protein | B8C991 | THAPSDRAFT_8355 | 12.7 | 4.4 |  | 2 | 3.7 | 22.5 | 22.5 | | Putative uncharacterized protein | B8BQL7 | THAPSDRAFT_1326 | 45.4 | 5.1 | sulphate assimilation | 2 | 3.7 | 4.6 | 4.6 | | Cold-shock DNA-binding domain-containing protein | B8C1Y0 | THAPSDRAFT_262414 | 7.8 | 4.4 | transcription regulation | 2 | 3.7 | 31.9 | 31.9 | | Ornithine cyclodeaminase | B8BQZ0 | THAPSDRAFT_260953 | 37.3 | 5.3 | metabolic process | 2 | 3.6 | 7.8 | 7.8 | | Inorganic pyrophosphatase | B8C6T9 | THAPSDRAFT_269348 | 30.0 | 4.8 | metabolic process | 2 | 3.6 | 10.3 | 10.3 | | Predicted protein | B8C0M3 | THAPSDRAFT_22483 | 152.9 | 4.9 |  | 2 | 3.5 | 2.2 | 2.2 | | RL14, ribosomal protein 14 | B8BUA7 | RL14 | 15.0 | 10.1 | translation | 2 | 3.5 | 17.9 | 17.9 | | RS25, ribosomal protein 25 | B8CCQ5 | RS25 | 12.6 | 10.1 | translation | 2 | 3.5 | 25.0 | 25.0 | | Fucoxanthin chlorophyll a/c light-harvesting protein | B8C2Y4 | Lhcr10 | 23.4 | 6.4 | photosynthesis | 2 | 3.5 | 12.7 | 12.7 | | Proteasome subunit alpha type | B8C0E7 | PSA4 | 29.4 | 4.9 | ubiquitin-dependent protein catabolic process | 2 | 3.5 | 8.5 | 8.5 | | Predicted protein | B8BZ42 | THAPSDRAFT_22214 | 11.4 | 4.5 |  | 2 | 3.5 | 28.3 | 24.5 | | Predicted protein | B8LCI4 | THAPSDRAFT_25130 | 50.1 | 6.3 | metabolic process | 2 | 3.4 | 6.4 | 6.4 | | Predicted protein | B8CCH4 | THAPSDRAFT_24864 | 29.7 | 5.5 | ion transport | 2 | 3.4 | 9.5 | 9.5 | | Actin-like protein | B8CFV4 | ACT1 | 41.8 | 5.0 | cytoskeleton | 2 | 3.4 | 9.0 | 9.0 | | Eukaryotic translation initiation factor 4A | B8CC33 | THAPSDRAFT_9716 | 42.4 | 5.5 | protein biosynthesis | 2 | 3.2 | 6.0 | 6.0 | | Predicted protein | B8C0D8 | THAPSDRAFT_4819 | 44.2 | 5.1 |  | 2 | 5.2 | 12.3 | 7.7 | | Photosystem I P700 chlorophyll a apoprotein A1 | A0T0M8 | psaA | 83.6 | 7.1 | photosynthesis | 2 | 3.1 | 3.9 | 2.8 | | Proteasome subunit beta type | B8BZW7 | PSB6 | 21.8 | 6.1 | protein catabolic process | 2 | 3.1 | 10.6 | 5.8 | | Predicted protein | B8CED8 | THAPSDRAFT_10741 | 15.0 | 5.0 |  | 2 | 2.7 | 19.1 | 10.6 | | Predicted protein | B8C5C2 | THAPSDRAFT_23024 | 12.3 | 6.8 |  | 2 | 2.7 | 39.7 | 13.8 | | Fucoxanthin chlorophyll a/c protein, LI818 clade | B8CGG2 | Lhcx2 | 22.2 | 4.5 | photosynthesis | 2 | 2.4 | 12.4 | 8.6 | | Peptidyl-prolyl cis-trans isomerase | B8CDT8 | THAPSDRAFT_37976 | 17.1 | 8.5 | protein folding | 2 | 2.3 | 6.7 | 6.7 | | 30S ribosomal protein S3 | A0T0X9 | rps3 | 24.1 | 8.9 | translation | 2 | 2.3 | 11.2 | 5.1 | | Fucoxanthin chlorophyll a/c protein 5 | B8CEV5 | Lhcf5 | 21.5 | 5.0 | photosynthesis | 2 | 4.3 | 20.0 | 20.0 | | Predicted protein | B8CBI7 | THAPSDRAFT_29861 | 166.9 | 5.6 | oxidation reduction | 2 | 2.1 | 1.4 | 1.4 | | RL39, ribosomal protein 39 | B8BVE4 | RL39 | 6.2 | 12.2 | translation | 2 | 2.0 | 20.0 | 20.0 | | Predicted protein | B8LEQ9 | THAPSDRAFT_bd1313 | 7.4 | 4.9 | protein stabilization | 2 | 2.0 | 21.2 | 21.2 | | Acyl carrier protein | B8C103 | THAPSDRAFT_33574 | 9.1 | 4.1 | fatty acid biosynthesis | 2 | 2.0 | 21.4 | 21.4 | | RL22, ribosomal protein 22 | B8C5H1 | RL22 | 13.8 | 9.5 | translation | 2 | 1.8 | 18.0 | 10.7 |   ^a^ Protein information (accession numbers, gene names, MW, pI and gene ontology) was taken from the UniProtKB/Swiss-Prot database.  ^b^ Proteins were identified from MS/MS data and the ProteinPilot software. |  |
| --- | --- | --- | --- | --- | --- | --- | --- | --- | --- | --- | --- | --- | --- | --- | --- | --- | --- | --- | --- | --- | --- | --- | --- | --- | --- | --- | --- | --- | --- | --- | --- | --- | --- | --- | --- | --- | --- | --- | --- | --- | --- | --- | --- | --- | --- | --- | --- | --- | --- | --- | --- | --- | --- | --- | --- | --- | --- | --- | --- | --- | --- | --- | --- | --- | --- | --- | --- | --- | --- | --- | --- | --- | --- | --- | --- | --- | --- | --- | --- | --- | --- | --- | --- | --- | --- | --- | --- | --- | --- | --- | --- | --- | --- | --- | --- | --- | --- | --- | --- | --- | --- | --- | --- | --- | --- | --- | --- | --- | --- | --- | --- | --- | --- | --- | --- | --- | --- | --- | --- | --- | --- | --- | --- | --- | --- | --- | --- | --- | --- | --- | --- | --- | --- | --- | --- | --- | --- | --- | --- | --- | --- | --- | --- | --- | --- | --- | --- | --- | --- | --- | --- | --- | --- | --- | --- | --- | --- | --- | --- | --- | --- | --- | --- | --- | --- | --- | --- | --- | --- | --- | --- | --- | --- | --- | --- | --- | --- | --- | --- | --- | --- | --- | --- | --- | --- | --- | --- | --- | --- | --- | --- | --- | --- | --- | --- | --- | --- | --- | --- | --- | --- | --- | --- | --- | --- | --- | --- | --- | --- | --- | --- | --- | --- | --- | --- | --- | --- | --- | --- | --- | --- | --- | --- | --- | --- | --- | --- | --- | --- | --- | --- | --- | --- | --- | --- | --- | --- | --- | --- | --- | --- | --- | --- | --- | --- | --- | --- | --- | --- | --- | --- | --- | --- | --- | --- | --- | --- | --- | --- | --- | --- | --- | --- | --- | --- | --- | --- | --- | --- | --- | --- | --- | --- | --- | --- | --- | --- | --- | --- | --- | --- | --- | --- | --- | --- | --- | --- | --- | --- | --- | --- | --- | --- | --- | --- | --- | --- | --- | --- | --- | --- | --- | --- | --- | --- | --- | --- | --- | --- | --- | --- | --- | --- | --- | --- | --- | --- | --- | --- | --- | --- | --- | --- | --- | --- | --- | --- | --- | --- | --- | --- | --- | --- | --- | --- | --- | --- | --- | --- | --- | --- | --- | --- | --- | --- | --- | --- | --- | --- | --- | --- | --- | --- | --- | --- | --- | --- | --- | --- | --- | --- | --- | --- | --- | --- | --- | --- | --- | --- | --- | --- | --- | --- | --- | --- | --- | --- | --- | --- | --- | --- | --- | --- | --- | --- | --- | --- | --- | --- | --- | --- | --- | --- | --- | --- | --- | --- | --- | --- | --- | --- | --- | --- | --- | --- | --- | --- | --- | --- | --- | --- | --- | --- | --- | --- | --- | --- | --- | --- | --- | --- | --- | --- | --- | --- | --- | --- | --- | --- | --- | --- | --- | --- | --- | --- | --- | --- | --- | --- | --- | --- | --- | --- | --- | --- | --- | --- | --- | --- | --- | --- | --- | --- | --- | --- | --- | --- | --- | --- | --- | --- | --- | --- | --- | --- | --- | --- | --- | --- | --- | --- | --- | --- | --- | --- | --- | --- | --- | --- | --- | --- | --- | --- | --- | --- | --- | --- | --- | --- | --- | --- | --- | --- | --- | --- | --- | --- | --- | --- | --- | --- | --- | --- | --- | --- | --- | --- | --- | --- | --- | --- | --- | --- | --- | --- | --- | --- | --- | --- | --- | --- | --- | --- | --- | --- | --- | --- | --- | --- | --- | --- | --- | --- | --- | --- | --- | --- | --- | --- | --- | --- | --- | --- | --- | --- | --- | --- | --- | --- | --- | --- | --- | --- | --- | --- | --- | --- | --- | --- | --- | --- | --- | --- | --- | --- | --- | --- | --- | --- | --- | --- | --- | --- | --- | --- | --- | --- | --- | --- | --- | --- | --- | --- | --- | --- | --- | --- | --- | --- | --- | --- | --- | --- | --- | --- | --- | --- | --- | --- | --- | --- | --- | --- | --- | --- | --- | --- | --- | --- | --- | --- | --- | --- | --- | --- | --- | --- | --- | --- | --- | --- | --- | --- | --- | --- | --- | --- | --- | --- | --- | --- | --- | --- | --- | --- | --- | --- | --- | --- | --- | --- | --- | --- | --- | --- | --- | --- | --- | --- | --- | --- | --- | --- | --- | --- | --- | --- | --- | --- | --- | --- | --- | --- | --- | --- | --- | --- | --- | --- | --- | --- | --- | --- | --- | --- | --- | --- | --- | --- | --- | --- | --- | --- | --- | --- | --- | --- | --- | --- | --- | --- | --- | --- | --- | --- | --- | --- | --- | --- | --- | --- | --- | --- | --- | --- | --- | --- | --- | --- | --- | --- | --- | --- | --- | --- | --- | --- | --- | --- | --- | --- | --- | --- | --- | --- | --- | --- | --- | --- | --- | --- | --- | --- | --- | --- | --- | --- | --- | --- | --- | --- | --- | --- | --- | --- | --- | --- | --- | --- | --- | --- | --- | --- | --- | --- | --- | --- | --- | --- | --- | --- | --- | --- | --- | --- | --- | --- | --- | --- | --- | --- | --- | --- | --- | --- | --- | --- | --- | --- | --- | --- | --- | --- | --- | --- | --- | --- | --- | --- | --- | --- | --- | --- | --- | --- | --- | --- | --- | --- | --- | --- | --- | --- | --- | --- | --- | --- | --- | --- | --- | --- | --- | --- | --- | --- | --- | --- | --- | --- | --- | --- | --- | --- | --- | --- | --- | --- | --- | --- | --- | --- | --- | --- | --- | --- | --- | --- | --- | --- | --- | --- | --- | --- | --- | --- | --- | --- | --- | --- | --- | --- | --- | --- | --- | --- | --- | --- | --- | --- | --- | --- | --- | --- | --- | --- | --- | --- | --- | --- | --- | --- | --- | --- | --- | --- | --- | --- | --- | --- | --- | --- | --- | --- | --- | --- | --- | --- | --- | --- | --- | --- | --- | --- | --- | --- | --- | --- | --- | --- | --- | --- | --- | --- | --- | --- | --- | --- | --- | --- | --- | --- | --- | --- | --- | --- | --- | --- | --- | --- | --- | --- | --- | --- | --- | --- | --- | --- | --- | --- | --- | --- | --- | --- | --- | --- | --- | --- | --- | --- | --- | --- | --- | --- | --- | --- | --- | --- | --- | --- | --- | --- | --- | --- | --- | --- | --- | --- | --- | --- | --- | --- | --- | --- | --- | --- | --- | --- | --- | --- | --- | --- | --- | --- | --- | --- | --- | --- | --- | --- | --- | --- | --- | --- | --- | --- | --- | --- | --- | --- | --- | --- | --- | --- | --- | --- | --- | --- | --- | --- | --- | --- | --- | --- | --- | --- | --- | --- | --- | --- | --- | --- | --- | --- | --- | --- | --- | --- | --- | --- | --- | --- | --- | --- | --- | --- | --- | --- | --- | --- | --- | --- | --- | --- | --- | --- | --- | --- | --- | --- | --- | --- | --- | --- | --- | --- | --- | --- | --- | --- | --- | --- | --- | --- | --- | --- | --- | --- | --- | --- | --- | --- | --- | --- | --- | --- | --- | --- | --- | --- | --- | --- | --- | --- | --- | --- | --- | --- | --- | --- | --- | --- | --- | --- | --- | --- | --- | --- | --- | --- | --- | --- | --- | --- | --- | --- | --- | --- | --- | --- | --- | --- | --- | --- | --- | --- | --- | --- | --- | --- | --- | --- | --- | --- | --- | --- | --- | --- | --- | --- | --- | --- | --- | --- | --- | --- | --- | --- | --- | --- | --- | --- | --- | --- | --- | --- | --- | --- | --- | --- | --- | --- | --- | --- | --- | --- | --- | --- | --- | --- | --- | --- | --- | --- | --- | --- | --- | --- | --- | --- | --- | --- | --- | --- | --- | --- | --- | --- | --- | --- | --- | --- | --- | --- | --- | --- | --- | --- | --- | --- | --- | --- | --- | --- | --- | --- | --- | --- | --- | --- | --- | --- | --- | --- | --- | --- | --- | --- | --- | --- | --- | --- | --- | --- | --- | --- | --- | --- | --- | --- | --- | --- | --- | --- | --- | --- | --- | --- | --- | --- | --- | --- | --- | --- | --- | --- | --- | --- | --- | --- | --- | --- | --- | --- | --- | --- | --- | --- | --- | --- | --- | --- | --- | --- | --- | --- | --- | --- | --- | --- | --- | --- | --- | --- | --- | --- | --- | --- | --- | --- | --- | --- | --- | --- | --- | --- | --- | --- | --- | --- | --- | --- | --- | --- | --- | --- | --- | --- | --- | --- | --- | --- | --- | --- | --- | --- | --- | --- | --- | --- | --- | --- | --- | --- | --- | --- | --- | --- | --- | --- | --- | --- | --- | --- | --- | --- | --- | --- | --- | --- | --- | --- | --- | --- | --- | --- | --- | --- | --- | --- | --- | --- | --- | --- | --- | --- | --- | --- | --- | --- | --- | --- | --- | --- | --- | --- | --- | --- | --- | --- | --- | --- | --- | --- | --- | --- | --- | --- | --- | --- | --- | --- | --- | --- | --- | --- | --- | --- | --- | --- | --- | --- | --- | --- | --- | --- | --- | --- | --- | --- | --- | --- | --- | --- | --- | --- | --- | --- | --- | --- | --- | --- | --- | --- | --- | --- | --- | --- | --- | --- | --- | --- | --- | --- | --- | --- | --- | --- | --- | --- | --- | --- | --- | --- | --- | --- | --- | --- | --- | --- | --- | --- | --- | --- | --- | --- | --- | --- | --- | --- | --- | --- | --- | --- | --- | --- | --- | --- | --- | --- | --- | --- | --- | --- | --- | --- | --- | --- | --- | --- | --- | --- | --- | --- | --- | --- | --- | --- | --- | --- | --- | --- | --- | --- | --- | --- | --- | --- | --- | --- | --- | --- | --- | --- | --- | --- | --- | --- | --- | --- | --- | --- | --- | --- | --- | --- | --- | --- | --- | --- | --- | --- | --- | --- | --- | --- | --- | --- | --- | --- | --- | --- | --- | --- | --- | --- | --- | --- | --- | --- | --- | --- | --- | --- | --- | --- | --- | --- | --- | --- | --- | --- | --- | --- | --- | --- | --- | --- | --- | --- | --- | --- | --- | --- | --- | --- | --- | --- | --- | --- | --- | --- | --- | --- | --- | --- | --- | --- | --- | --- | --- | --- | --- | --- | --- | --- | --- | --- | --- | --- | --- | --- | --- | --- | --- | --- | --- | --- | --- | --- | --- | --- | --- | --- | --- | --- | --- | --- | --- | --- | --- | --- | --- | --- | --- | --- | --- | --- | --- | --- | --- | --- | --- | --- | --- | --- | --- | --- | --- | --- | --- | --- | --- | --- | --- | --- | --- | --- | --- | --- | --- | --- | --- | --- | --- | --- | --- | --- | --- | --- | --- | --- | --- | --- | --- | --- | --- | --- | --- | --- | --- | --- | --- | --- | --- | --- | --- | --- | --- | --- | --- | --- | --- | --- | --- | --- | --- | --- | --- | --- | --- | --- | --- | --- | --- | --- | --- | --- | --- | --- | --- | --- | --- | --- | --- | --- | --- | --- | --- | --- | --- | --- | --- | --- | --- | --- | --- | --- | --- | --- | --- | --- | --- | --- | --- | --- | --- | --- | --- | --- | --- | --- | --- | --- | --- | --- | --- | --- | --- | --- | --- | --- | --- | --- | --- | --- | --- | --- | --- | --- | --- | --- | --- | --- | --- | --- | --- | --- | --- | --- | --- | --- | --- | --- | --- | --- | --- | --- | --- | --- | --- | --- | --- | --- | --- | --- | --- | --- | --- | --- | --- | --- | --- | --- | --- | --- | --- | --- | --- | --- | --- | --- | --- | --- | --- | --- | --- | --- | --- | --- | --- | --- | --- | --- | --- | --- | --- | --- | --- | --- | --- | --- | --- | --- | --- | --- | --- | --- | --- | --- | --- | --- | --- | --- | --- | --- | --- | --- | --- | --- | --- | --- | --- | --- | --- | --- | --- | --- | --- | --- | --- | --- | --- | --- | --- | --- | --- | --- | --- | --- | --- | --- | --- | --- | --- | --- | --- | --- | --- | --- | --- | --- | --- | --- | --- | --- | --- | --- | --- | --- | --- | --- | --- | --- | --- | --- | --- | --- | --- | --- | --- | --- | --- | --- | --- | --- | --- | --- | --- | --- | --- | --- | --- | --- | --- | --- | --- | --- | --- | --- | --- | --- | --- | --- | --- | --- | --- | --- | --- | --- | --- | --- | --- | --- | --- | --- | --- | --- | --- | --- | --- | --- | --- | --- | --- | --- | --- | --- | --- | --- | --- | --- | --- | --- | --- | --- | --- | --- | --- | --- | --- | --- | --- | --- | --- | --- | --- | --- | --- | --- | --- | --- | --- | --- | --- | --- | --- | --- | --- | --- | --- | --- | --- | --- | --- | --- | --- | --- | --- | --- | --- | --- | --- | --- | --- | --- | --- | --- | --- | --- | --- | --- | --- | --- | --- | --- | --- | --- | --- | --- | --- | --- | --- | --- | --- | --- | --- | --- | --- | --- | --- | --- | --- | --- | --- | --- | --- | --- | --- | --- | --- | --- | --- | --- | --- | --- | --- | --- | --- | --- | --- | --- | --- | --- | --- | --- | --- | --- | --- | --- | --- | --- | --- | --- | --- | --- | --- | --- | --- | --- | --- | --- | --- | --- | --- | --- | --- | --- | --- | --- | --- | --- | --- | --- | --- | --- | --- | --- | --- | --- | --- | --- | --- | --- | --- | --- | --- | --- | --- | --- | --- | --- |
