# Supplementary material for: Proteomic analysis of the marine diatom Thalassiosira pseudonana upon exposure to benzo(a)pyrene
Source: BMC Genomics. 2011 Mar 24;12:159. doi: 10.1186/1471-2164-12-159 (PMC3076255; doi:10.1186/1471-2164-12-159)
Supplement: Additional file 4 — Peptide fragmentation spectra used for the identification and relative quantification of proteins that have been included in Table 1as potentially regulated. The file contains the fragmentation spectra of the peptide fragment identified with high confidence (> 95%) as well as the fragmentation spectra of other peptides identified with lower confidence, for six different proteins (Supplemental Figures S1-S6). The reporter ion signals shown in the low-mass region of the spectra were used to determine the relative amount of the protein in T. pseudonana exposed to BaP (iTRAQ 114, 116 and 121) when compared to control conditions (iTRAQ 113, 115 and 119). [file 1471-2164-12-159-S4.PPT]

## Slide 1
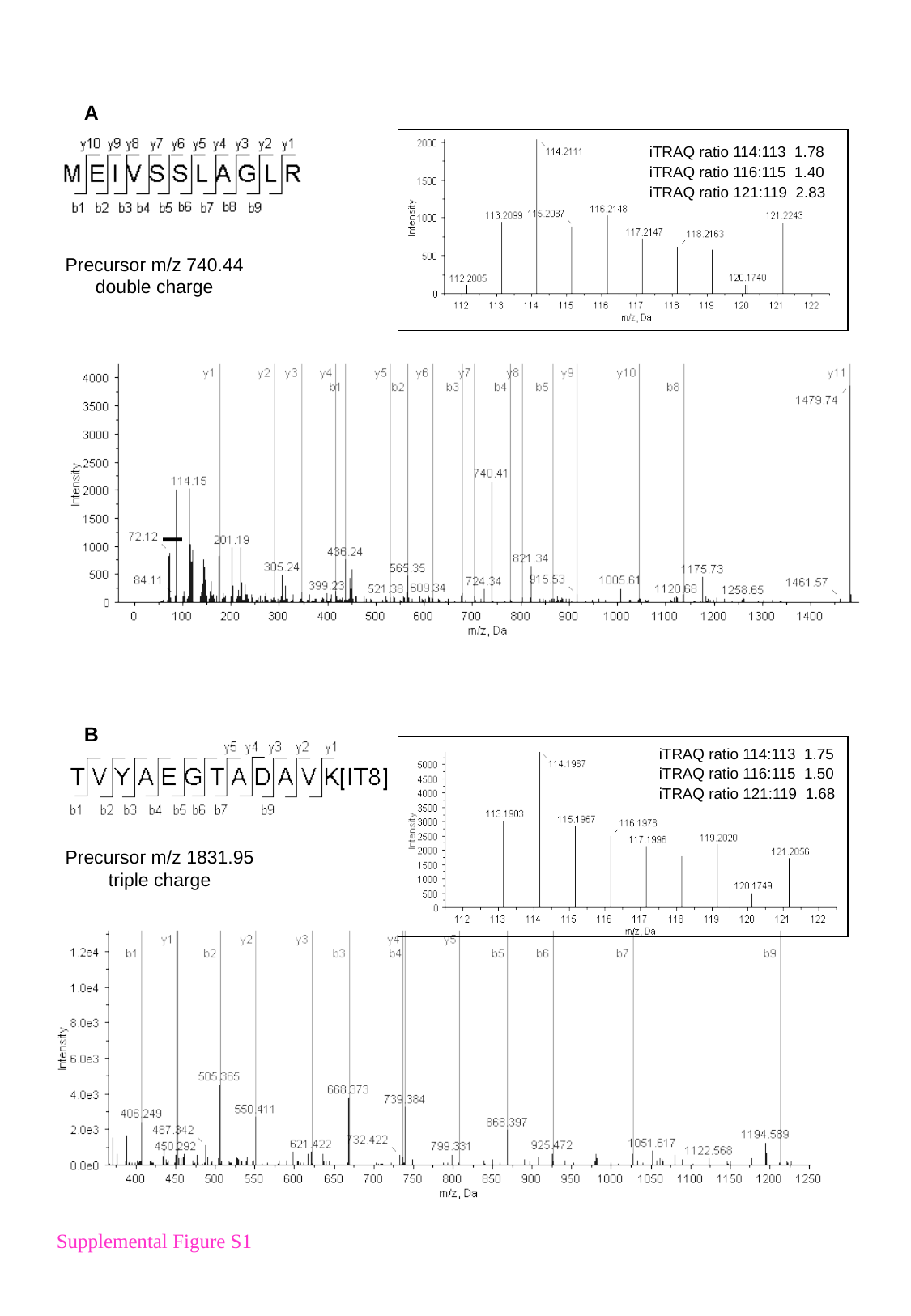

A
iTRAQ ratio 114:113 1.78
iTRAQ ratio 116:115 1.40
iTRAQ ratio 121:119 2.83
Precursor m/z 740.44
double charge
B
iTRAQ ratio 114:113 1.75
iTRAQ ratio 116:115 1.50
iTRAQ ratio 121:119 1.68
Precursor m/z 1831.95
triple charge
Supplemental Figure S1

## Slide 2
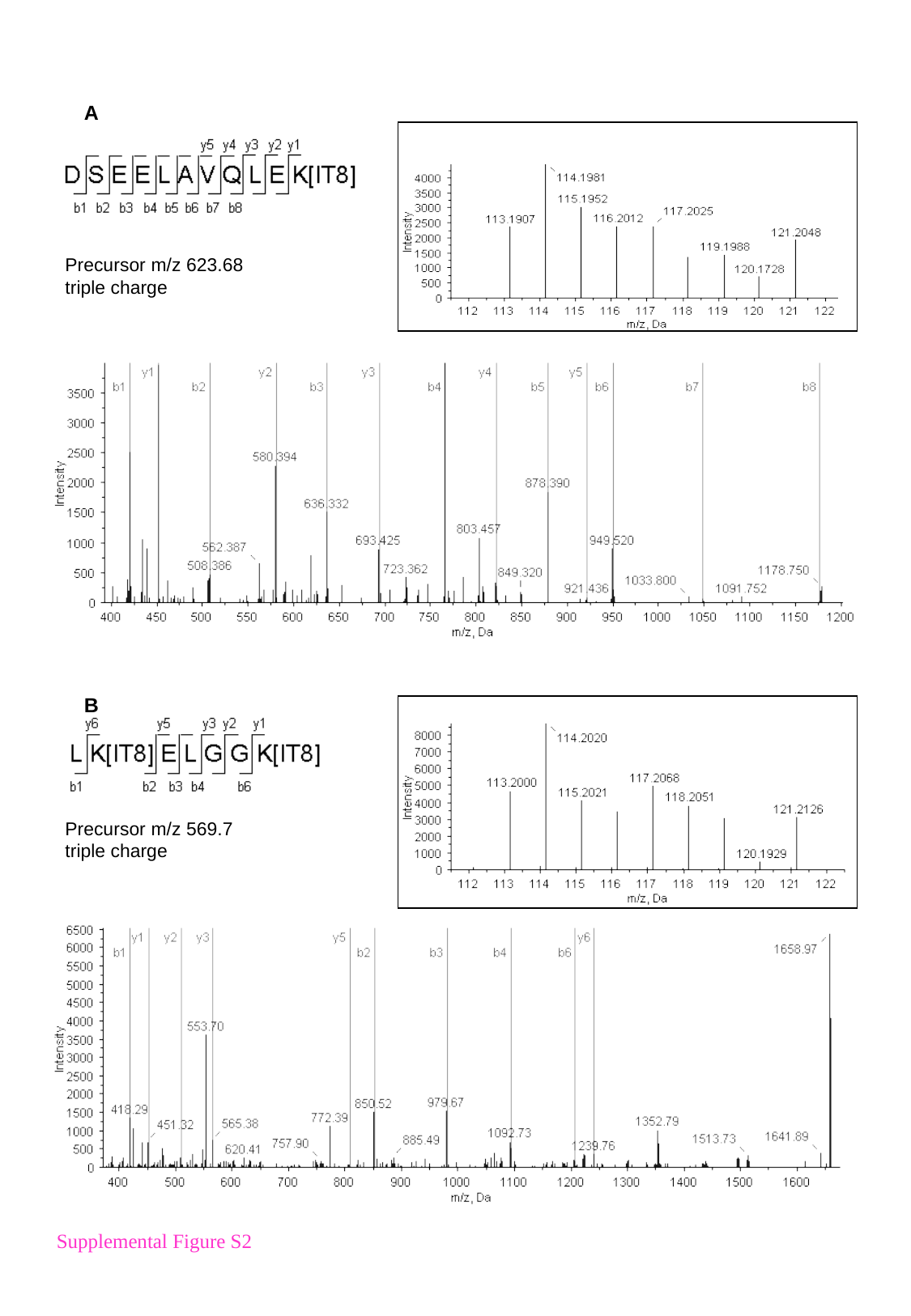

A
Precursor m/z 623.68
triple charge
B
Precursor m/z 569.7
triple charge
Supplemental Figure S2

## Slide 3
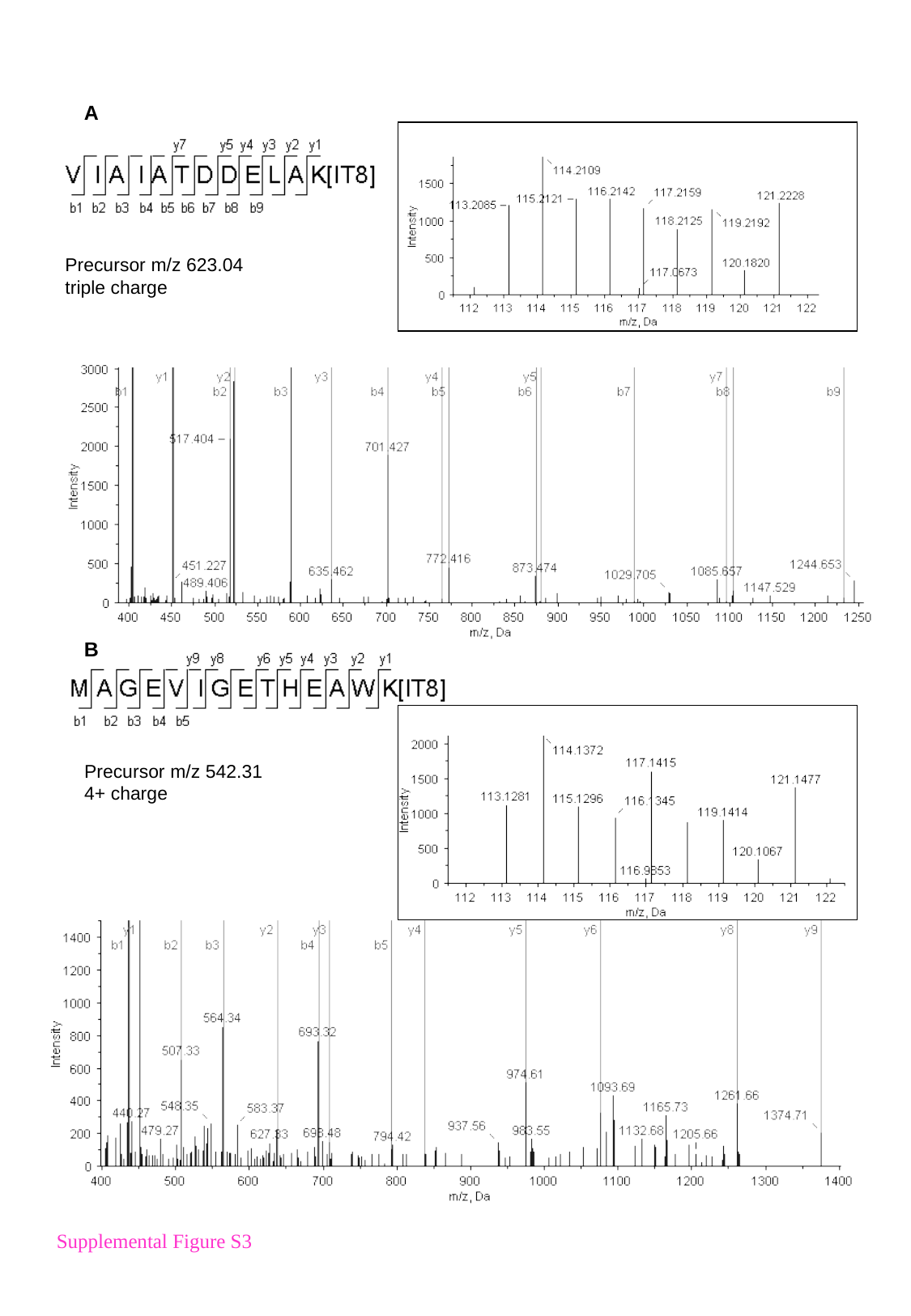

A
Precursor m/z 623.04
triple charge
B
Precursor m/z 542.31
4+ charge
Supplemental Figure S3

## Slide 4
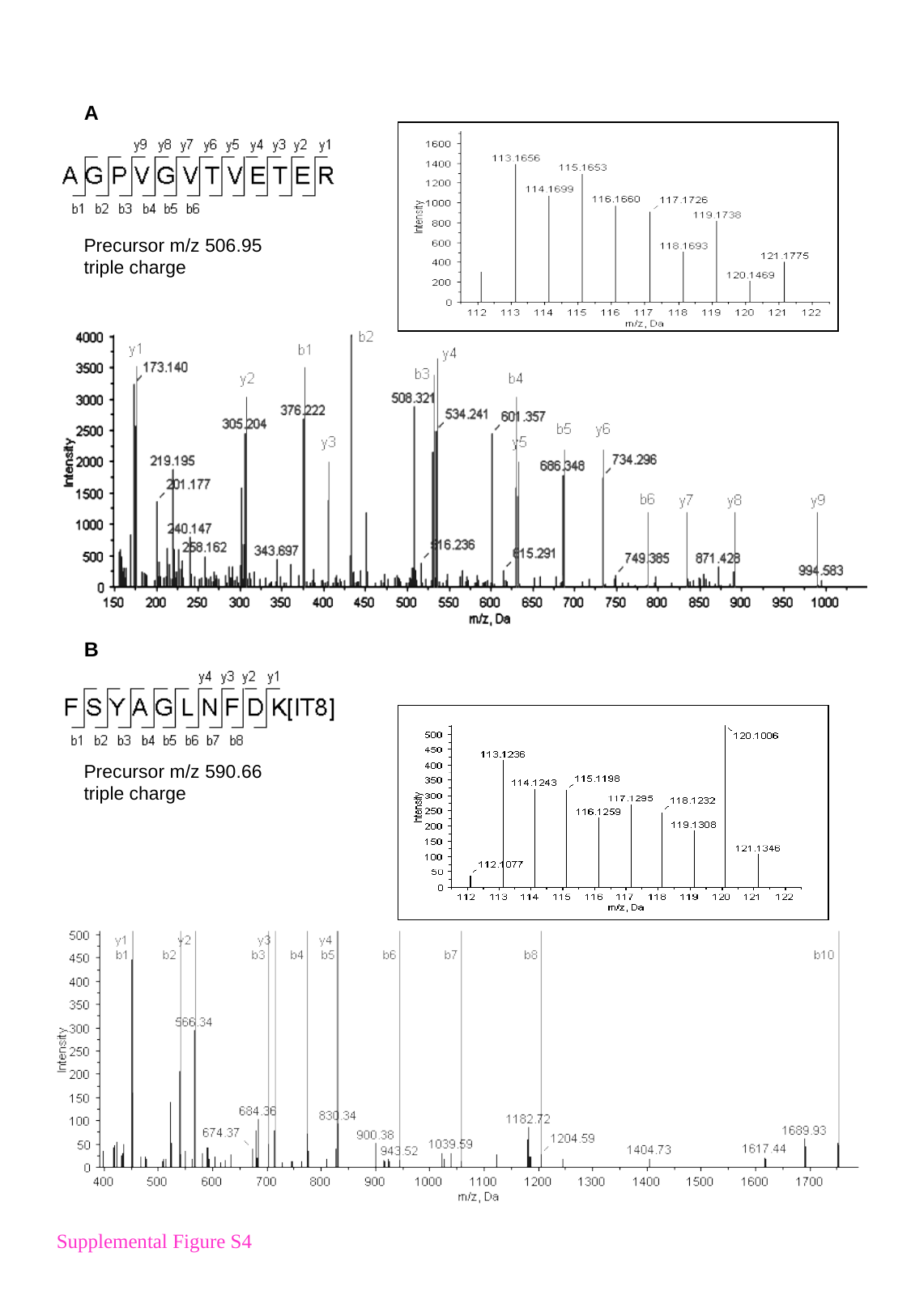

A
Precursor m/z 506.95
triple charge
B
Precursor m/z 590.66
triple charge
Supplemental Figure S4

## Slide 5
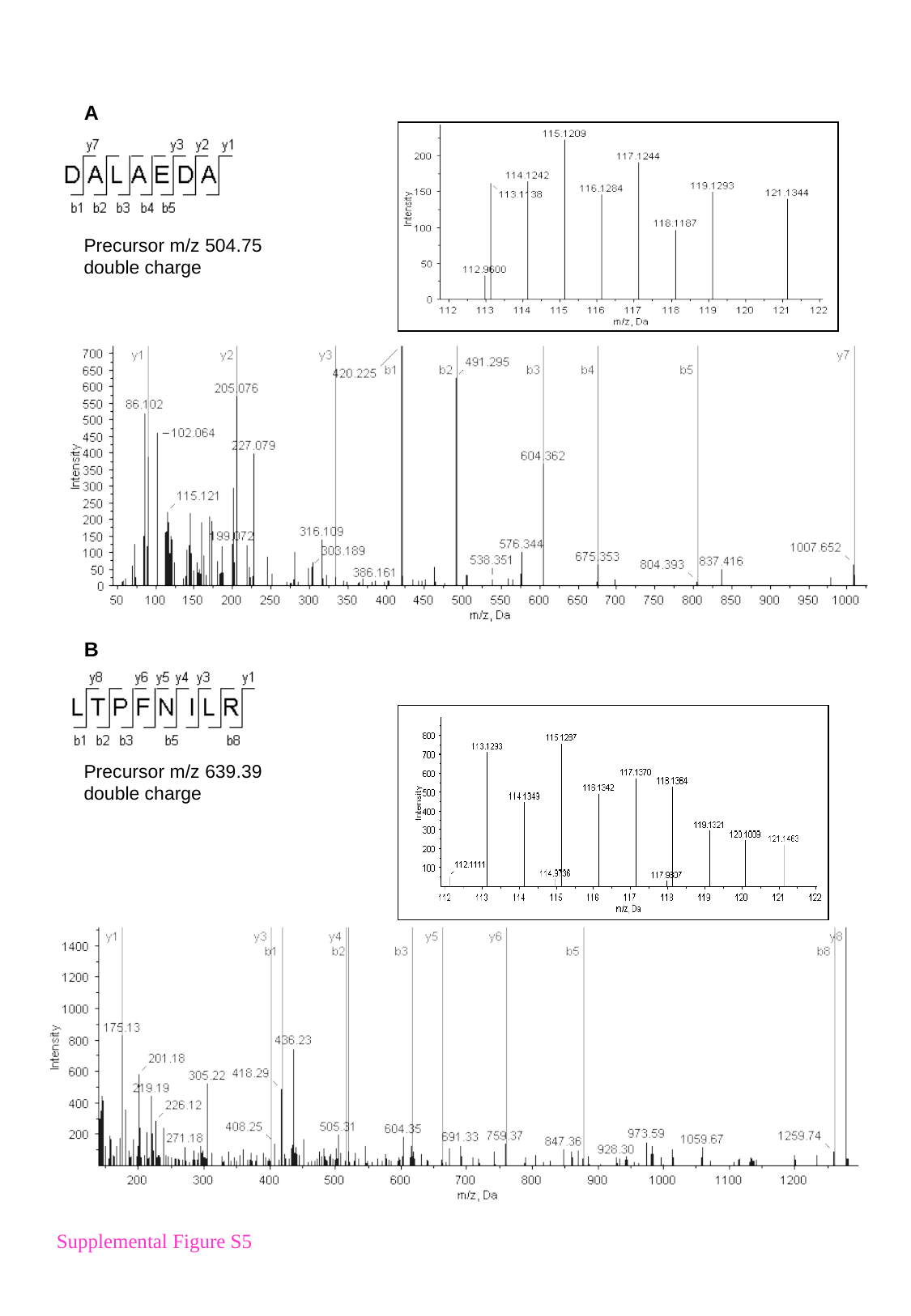

A
Precursor m/z 504.75
double charge
B
Precursor m/z 639.39
double charge
Supplemental Figure S5

## Slide 6
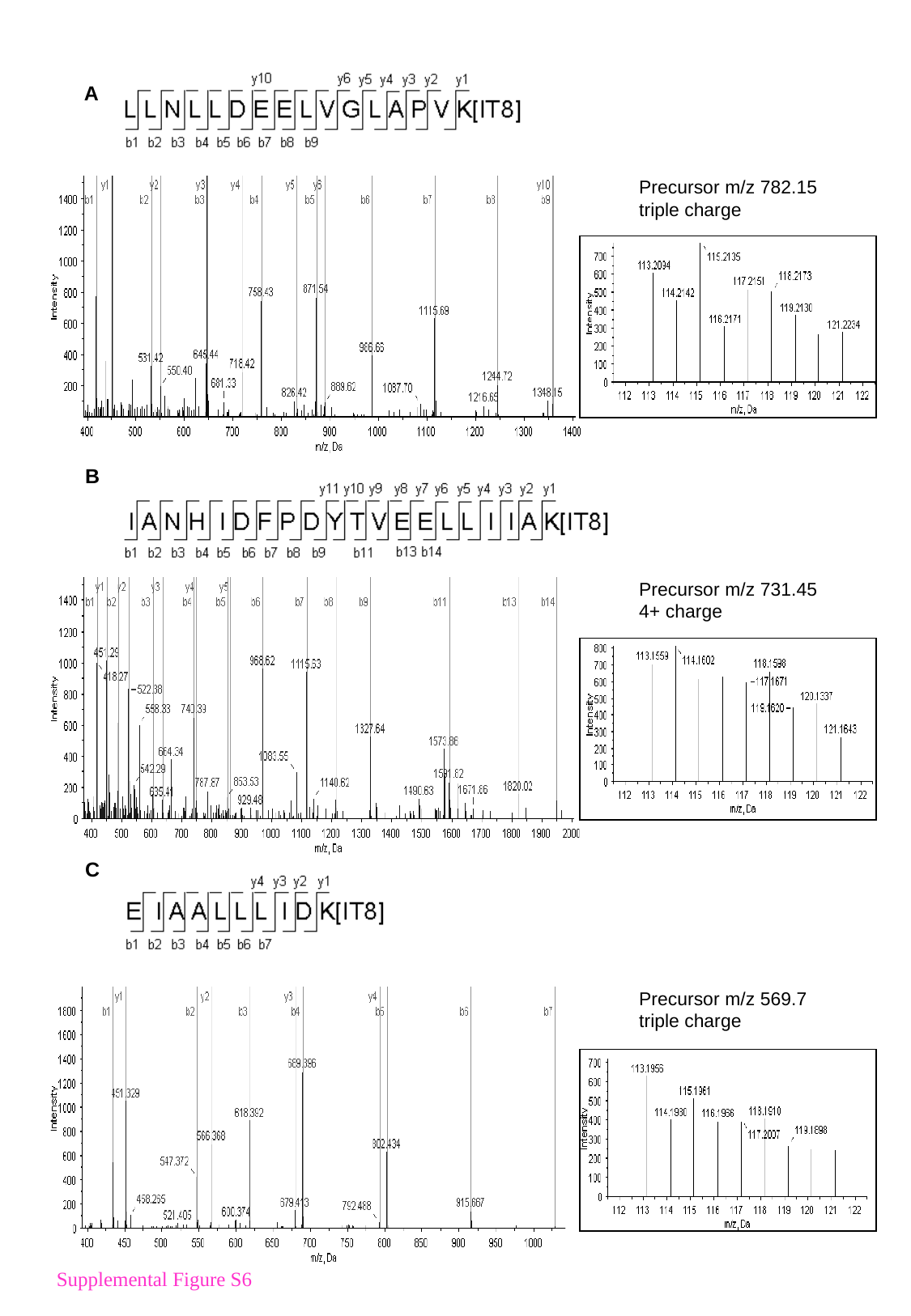

A
Precursor m/z 782.15
triple charge
B
Precursor m/z 731.45
4+ charge
C
Precursor m/z 569.7
triple charge
Supplemental Figure S6

## Slide 7
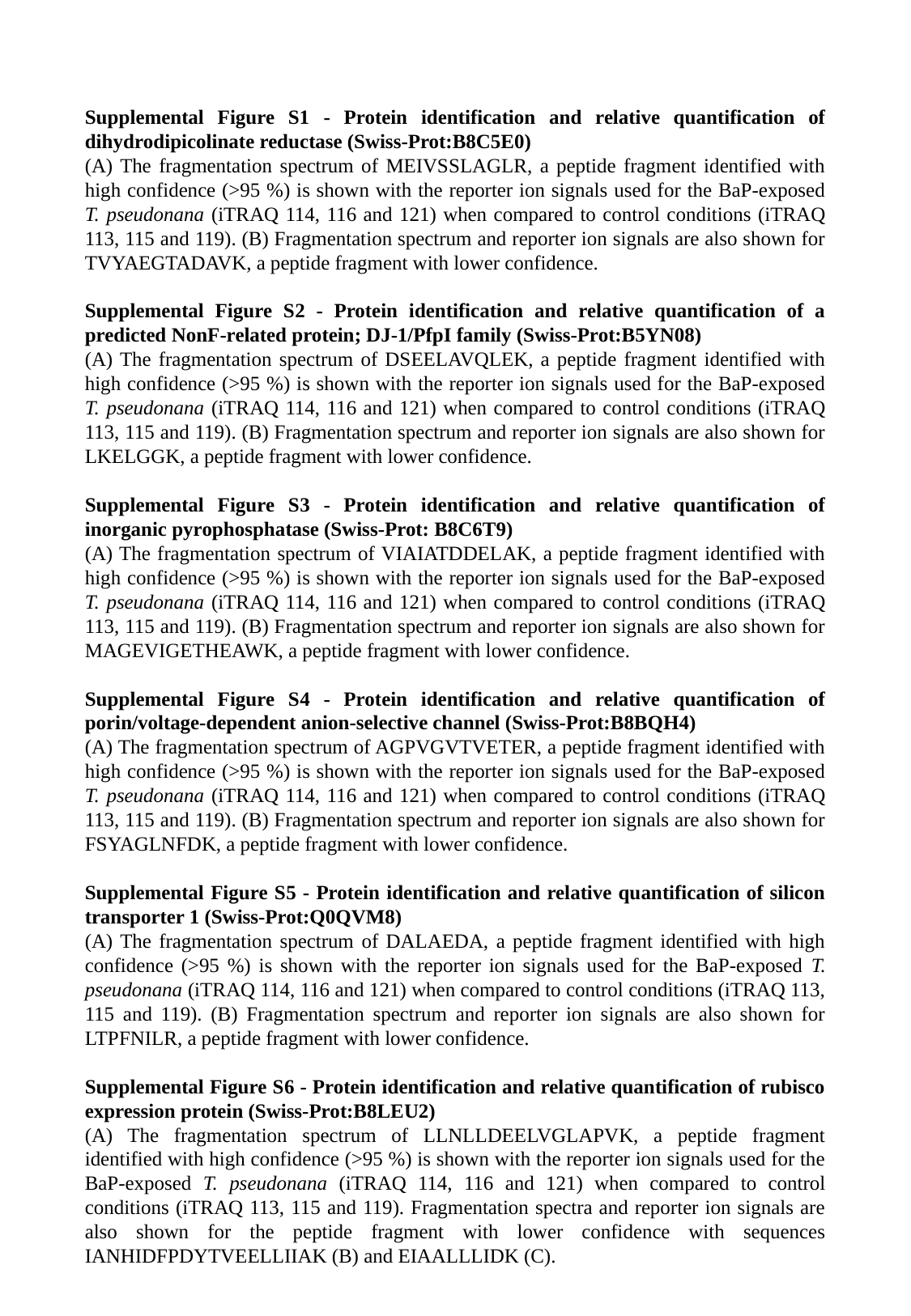

Supplemental Figure S1 - Protein identification and relative quantification of dihydrodipicolinate reductase (Swiss-Prot:B8C5E0)
(A) The fragmentation spectrum of MEIVSSLAGLR, a peptide fragment identified with high confidence (>95 %) is shown with the reporter ion signals used for the BaP-exposed T. pseudonana (iTRAQ 114, 116 and 121) when compared to control conditions (iTRAQ 113, 115 and 119). (B) Fragmentation spectrum and reporter ion signals are also shown for TVYAEGTADAVK, a peptide fragment with lower confidence.
Supplemental Figure S2 - Protein identification and relative quantification of a predicted NonF-related protein; DJ-1/PfpI family (Swiss-Prot:B5YN08)
(A) The fragmentation spectrum of DSEELAVQLEK, a peptide fragment identified with high confidence (>95 %) is shown with the reporter ion signals used for the BaP-exposed T. pseudonana (iTRAQ 114, 116 and 121) when compared to control conditions (iTRAQ 113, 115 and 119). (B) Fragmentation spectrum and reporter ion signals are also shown for LKELGGK, a peptide fragment with lower confidence.
Supplemental Figure S3 - Protein identification and relative quantification of inorganic pyrophosphatase (Swiss-Prot: B8C6T9)
(A) The fragmentation spectrum of VIAIATDDELAK, a peptide fragment identified with high confidence (>95 %) is shown with the reporter ion signals used for the BaP-exposed T. pseudonana (iTRAQ 114, 116 and 121) when compared to control conditions (iTRAQ 113, 115 and 119). (B) Fragmentation spectrum and reporter ion signals are also shown for MAGEVIGETHEAWK, a peptide fragment with lower confidence.
Supplemental Figure S4 - Protein identification and relative quantification of porin/voltage-dependent anion-selective channel (Swiss-Prot:B8BQH4)
(A) The fragmentation spectrum of AGPVGVTVETER, a peptide fragment identified with high confidence (>95 %) is shown with the reporter ion signals used for the BaP-exposed T. pseudonana (iTRAQ 114, 116 and 121) when compared to control conditions (iTRAQ 113, 115 and 119). (B) Fragmentation spectrum and reporter ion signals are also shown for FSYAGLNFDK, a peptide fragment with lower confidence.
Supplemental Figure S5 - Protein identification and relative quantification of silicon transporter 1 (Swiss-Prot:Q0QVM8)
(A) The fragmentation spectrum of DALAEDA, a peptide fragment identified with high confidence (>95 %) is shown with the reporter ion signals used for the BaP-exposed T. pseudonana (iTRAQ 114, 116 and 121) when compared to control conditions (iTRAQ 113, 115 and 119). (B) Fragmentation spectrum and reporter ion signals are also shown for LTPFNILR, a peptide fragment with lower confidence.
Supplemental Figure S6 - Protein identification and relative quantification of rubisco expression protein (Swiss-Prot:B8LEU2)
(A) The fragmentation spectrum of LLNLLDEELVGLAPVK, a peptide fragment identified with high confidence (>95 %) is shown with the reporter ion signals used for the BaP-exposed T. pseudonana (iTRAQ 114, 116 and 121) when compared to control conditions (iTRAQ 113, 115 and 119). Fragmentation spectra and reporter ion signals are also shown for the peptide fragment with lower confidence with sequences IANHIDFPDYTVEELLIIAK (B) and EIAALLLIDK (C).
