# Supplementary material for: Proteomic analysis of the marine diatom Thalassiosira pseudonana upon exposure to benzo(a)pyrene
Source: BMC Genomics. 2011 Mar 24;12:159. doi: 10.1186/1471-2164-12-159 (PMC3076255; doi:10.1186/1471-2164-12-159)
Supplement: Additional file 5 — Principle component analysis for the quantification of the iTRAQ labels in the different experiments. The analysis shows a clustering of the proteome analysis from control diatom cultures, labeled with iTRAQ 113, 115 and 119, as well as a clustering of the proteome analysis from the BaP-exposed conditions, labeled with iTRAQ 114, 116 and 121. An additional experiment has been incorporated in the MS/MS analysis, consisting of control and BaP-exposed conditions, labeled with iTRAQ 117 and 118, respectively, but were not considered for the overall quantification analysis. In this experiment, the same starting density of 0.5 million cells/mL was used, but from a culture in the resting phase. We observed that the growth in the diatom cultures was largely reduced from the expected values, and was also observed after the addition of solvent or BaP. We decided to use the cells arising from this experiment with the remaining iTRAQ labels from the 8-plex kit, to see whether under these abnormal cell culture circumstances, we could still observe significant differences between control and exposure conditions. It is evident that both conditions in this experiment sit as outliers on the graph and in addition, they seem to be clustered together. In fact, from the quantification analysis, it was observed that both 117 and 118 labels showed rather consistent values for most of the peptides analyzed, and not in agreement with the experimental conditions, i.e. control or BaP-exposed. It is interesting to note that the reduced cell growth in this culture, greatly affected the outcome of the quantification. Thus, the apparent sensitivity of these cells seemed to be more relevant than the exposure conditions themselves. These findings, although not unexpected, truly support the importance of reproducibility in biomarker research, since any differences in the initial state of the cells has a substantial effect on the biomolecular pool and thus masks any potential differences of expressio [file 1471-2164-12-159-S5.PPT]

## Slide 1
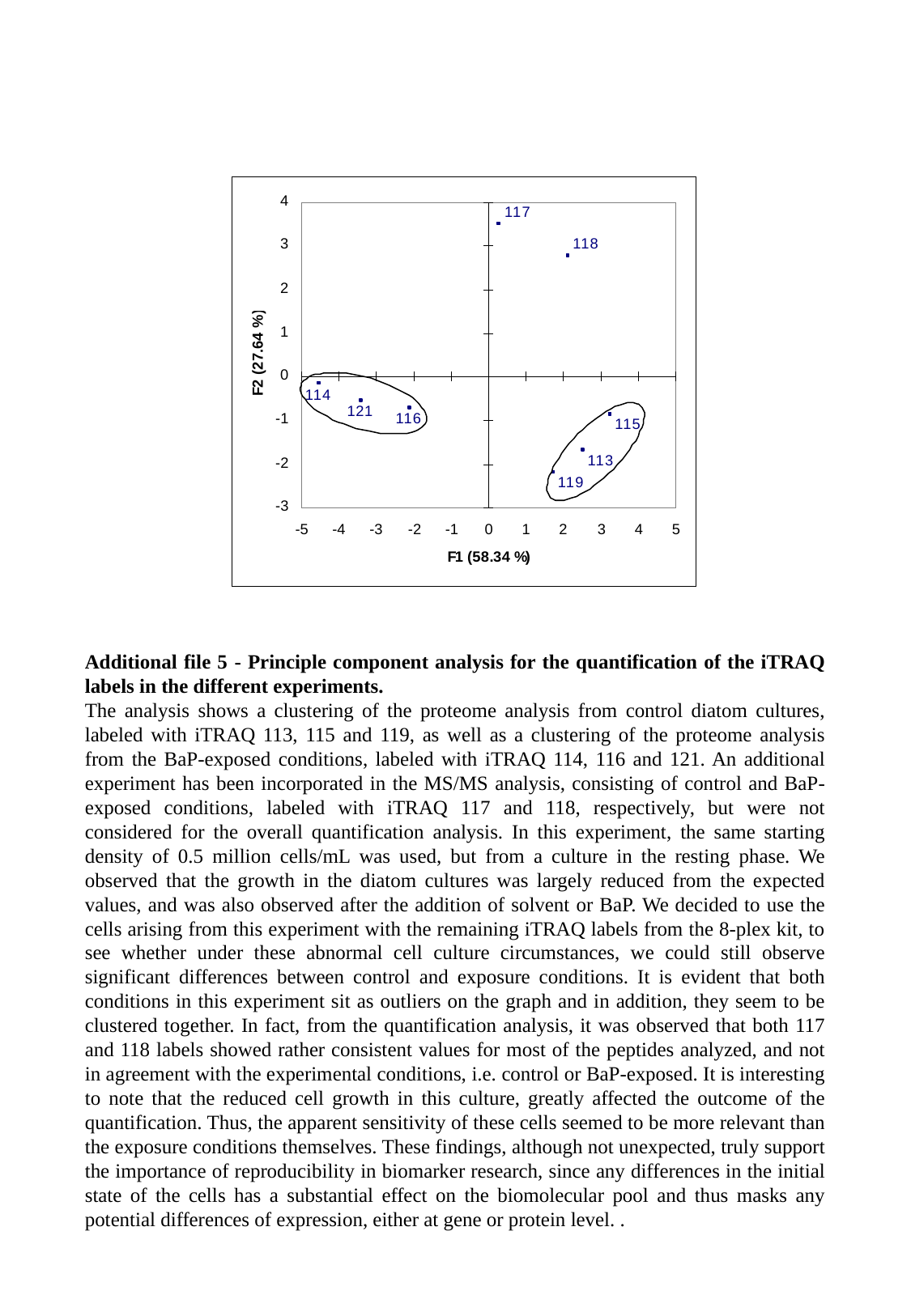

Additional file 5 - Principle component analysis for the quantification of the iTRAQ labels in the different experiments.
The analysis shows a clustering of the proteome analysis from control diatom cultures, labeled with iTRAQ 113, 115 and 119, as well as a clustering of the proteome analysis from the BaP-exposed conditions, labeled with iTRAQ 114, 116 and 121. An additional experiment has been incorporated in the MS/MS analysis, consisting of control and BaP-exposed conditions, labeled with iTRAQ 117 and 118, respectively, but were not considered for the overall quantification analysis. In this experiment, the same starting density of 0.5 million cells/mL was used, but from a culture in the resting phase. We observed that the growth in the diatom cultures was largely reduced from the expected values, and was also observed after the addition of solvent or BaP. We decided to use the cells arising from this experiment with the remaining iTRAQ labels from the 8-plex kit, to see whether under these abnormal cell culture circumstances, we could still observe significant differences between control and exposure conditions. It is evident that both conditions in this experiment sit as outliers on the graph and in addition, they seem to be clustered together. In fact, from the quantification analysis, it was observed that both 117 and 118 labels showed rather consistent values for most of the peptides analyzed, and not in agreement with the experimental conditions, i.e. control or BaP-exposed. It is interesting to note that the reduced cell growth in this culture, greatly affected the outcome of the quantification. Thus, the apparent sensitivity of these cells seemed to be more relevant than the exposure conditions themselves. These findings, although not unexpected, truly support the importance of reproducibility in biomarker research, since any differences in the initial state of the cells has a substantial effect on the biomolecular pool and thus masks any potential differences of expression, either at gene or protein level. .
